# Pollinator sharing between reproductively isolated genetic lineages of *Silene nutans*

**DOI:** 10.3389/fpls.2022.927498

**Published:** 2022-10-21

**Authors:** Camille Cornet, Nausicaa Noret, Fabienne Van Rossum

**Affiliations:** ^1^ Laboratoire d’Ecologie végétale et Biogéochimie, Université libre de Bruxelles, Brussels, Belgium; ^2^ Research Department, Meise Botanic Garden, Meise, Belgium; ^3^ Service général de l’Enseignement supérieur et de la Recherche scientifique, Fédération Wallonie-Bruxelles, Brussels, Belgium

**Keywords:** facilitation, fluorescent dye, nocturnal moths, nursery pollinators, pollen dispersal, reproductive isolation, seed predator, *Silene nutans*

## Abstract

High reciprocal pollination specialization leading to pollinator isolation can prevent interspecific pollen transfer and competition for pollinators. Sharing pollinators may induce mating costs, but it may also increase pollination services and pollen dispersal and offer more resources to pollinators, which may be important in case of habitat fragmentation leading to pollination disruption. We estimated pollen dispersal and pollinator isolation or sharing between two reproductively isolated genetic lineages of *Silene nutans* (Caryophyllaceae), which are rare and occur in parapatry in southern Belgium, forming two edaphic ecotypes. As inter-ecotypic crosses may lead to pollen wastage and inviable progeny, pollinator isolation might have evolved between ecotypes. *Silene nutans* is mainly pollinated by nocturnal moths, including nursery pollinators, which pollinate and lay their eggs in flowers, and whose caterpillars feed on flowers and seeds. Pollinator assemblages of the two ecotypes are largely unknown and inter-ecotypic pollen flows have never been investigated. Fluorescent powdered dyes were used as pollen analogues to quantify intra- and inter-ecotypic pollen transfers and seeds were germinated to detect chlorotic seedlings resulting from inter-ecotypic pollination. Nocturnal pollinators were observed using infrared cameras on the field, and seed-eating caterpillars were collected and reared to identify nursery pollinator species. No pollinator isolation was found: we detected long-distance (up to 5 km) inter-ecotypic dye transfers and chlorotic seedlings, indicating inter-ecotypic fertilization events. The rare moth *Hadena albimacula*, a nursery pollinator specialized on *S. nutans*, was found on both ecotypes, as well as adults visiting flowers (cameras recordings) as seed-eating caterpillars. However, *S. nutans* populations harbor different abundance and diversity of seed predator communities, including other rare nursery pollinators, suggesting a need for distinct conservation strategies. Our findings demonstrate the efficiency of moths, especially of nursery pollinators, to disperse pollen over long distances in natural landscapes, so to ensure gene flow and population sustainability of the host plant. Seed-predator specificities between the two reproductively isolated genetic lineages of *S. nutans*, and pollinator sharing instead of pollinator isolation when plants occur in parapatry, suggest that conservation of the host plant is also essential for sustaining (rare) pollinator and seed predator communities.

## Introduction

Plant-pollinator interactions represent an important relationship for plants that depend on pollinators for successful sexual reproduction and gene flow, while pollinators rely on resources provided by plants (nectar and pollen) (e.g., Kearns et al., 1998). Specialized pollinators may also depend on the pollinated plant species for their own reproduction, such as in nursery pollination (Hossaert-McKey et al., 2010). In nursery pollination, insects do not only pollinate but also lay eggs in flowers, the larvae subsequently feeding on flowers or seeds (Hembry and Althoff, 2016). The fig/fig wasps and yucca/yucca moths systems are well-known examples (Pellmyr, 2003; Herre et al., 2008) in which both insects and plants obligatory depend on each other for reproduction (Hembry and Althoff, 2016). Other systems, such as the *Silene*/*Hadena* system, can be more generalist: the nursery pollinator coexists with other pollinators, and *Hadena* moths (Noctuidae) are able to use several *Silene* species as host plants, themselves having several *Hadena* species as efficient pollinators (Kephart et al., 2006; Prieto-Benítez et al., 2017).

High reciprocal pollination specialization can have advantages for plant reproduction, e.g. preventing interspecific pollen transfer and competition for pollinators (Moreira-Hernández and Muchhala, 2019; Phillips et al., 2020). Furthermore, sharing pollinators can induce costs. Interspecific pollen transfer can result in pollen wastage (Inouye et al., 1994). In case of closely related species or diverging genetic lineages with incomplete reproductive isolation, seed production may still result from crosses between species or lineages sharing pollinators (Kay and Schemske, 2008). The resulting hybrid progeny may be inviable or sterile (e.g. Moccia et al., 2007), and maladapted to parental habitats in case of ecological specialization (Melo et al., 2014; Cahenzli et al., 2018). Therefore, prezygotic reproductive isolation barriers may be favored, especially when species or lineages co-occur in parapatry or sympatry (Ramsey et al., 2003; Baack et al., 2015). As a consequence, temporal isolation through differences in phenology (Michalski and Durka, 2015), and/or pollinator isolation, i.e. distinct pollinator assemblages, may be favored (Okamoto et al., 2015; Ramírez-Aguirre et al., 2019). However, pollinator sharing might also represent an advantage through increasing pollinator services and facilitating pollen dispersal for both plant species or lineages (Ghazoul, 2006; Phillips et al., 2020), and through offering more resources to pollinators, also possibly over a longer period of time (Moeller, 2004). While high pollination specialization can be problematic in the context of habitat fragmentation leading to the decline of pollinators and to disruption of plant-pollinator interactions, sharing pollinators may contribute to maintain plant reproductive success (e.g. Moeller, 2004; Ha et al., 2021).

Whether closely related species show pollinator isolation or pollinator sharing has been investigated in several model groups, such as *Mimulus* (Ramsey et al., 2003), *Costus* (Kay and Schemske, 2003), *Gelsemium* (Pascarella, 2007), and *Iochroma* (Smith et al., 2008). In most studies, pollinator isolation has been inferred from observations of pollinator activity, not by directly studying pollen transfer. This can be misleading because pollinators might differ not only in identity and visitation rates, but also in pollen transfer efficiency (Kay and Sargent, 2009). Some studies have tried to reduce this bias, by combining visitation rates and pollen deposition by different pollinators to estimate pollinator importance (e.g. Smith et al., 2008). Another approach relies on the use of fluorescent powdered dyes as pollen analogues to model pollen dispersal, in addition to pollinator observations (Goulson and Jerrim, 1997). The use of fluorescent powdered dyes is a quite convenient method to mimic pollinator movements and can be a reliable estimator of pollen flow within and among populations (Van Rossum, 2010; Van Rossum et al., 2011; Mayer et al., 2012).

In the present study, we investigated pollinator isolation or sharing between two edaphic (i.e. related to soil conditions) ecotypes of *Silene nutans* L. (Caryophyllaceae). *Silene nutans* is characterized by nocturnal moth pollination and is involved in nursery pollination relationships (Hepper, 1956; Jürgens et al., 1996). In Europe, *S. nutans* consists of an assemblage of seven western and eastern distinct genetic lineages, which have diverged in allopatry in separate glacial refugia during Quaternary climate oscillations before a stepwise northward recolonization, with secondary contact zones in Western Europe (Martin et al., 2016; Van Rossum et al., 2018). In particular, the western genetic lineage W1 and the eastern genetic lineage E1 differ in morphological traits (e.g. in leaf shape, floral display, and capsule and seed size) and in phenology (flowering from late April to early July, with E1 lineage flowering earlier than W1, with a flowering overlap of approximately two weeks) (De Bilde, 1973; Van Rossum, 2000). Mechanisms of pre- and postzygotic reproductive isolation have been shown between these two genetic lineages, with pollen-stigma incompatibilities and hybrid inviability (chlorosis) and sterility (Van Rossum et al., 1996; Martin et al., 2017; Postel et al., 2022). Interestingly, in the secondary contact zone in southern Belgium, the W1 and E1 genetic lineages of *S. nutans* form two distinct ecotypes: a calcicolous ecotype on calcareous soils (E1 lineage) and a silicicolous ecotype on siliceous soils (W1 lineage) (De Bilde, 1973; Martin et al., 2016; Van Rossum et al., 2018). Both ecotypes occur as distinct parapatric populations, sometimes separated by short geographic distances (< 1 km; Van Rossum et al., 1997; Van Rossum et al., 1999).

The strong postzygotic isolation between ecotypes of *S. nutans* suggests that inter-ecotypic pollination might cause pollen losses or lead to mating costs, as investment in seeds produces chlorotic, inviable hybrids. Therefore, prezygotic isolation mechanisms might be selected for, such as divergence in flowering periods or in floral traits, e.g. flower color and size, scent and nectar composition, resulting in different visiting pollinator assemblages and in pollinator isolation (e.g. Schemske and Bradshaw, 1999; Waelti et al., 2008; Ramírez-Aguirre et al., 2019). Divergence in floral traits between *S. nutans* ecotypes, such as flower color (white for calcicolous plants and greenish, yellowish to pink for silicicolous plants) and size of the petal scale (1.9 times longer for the calcicolous ecotype; De Bilde, 1973), indeed suggests possible pollinator isolation. Additionally, pollinator isolation can more easily arise in case of pollinator specialization (Kay and Sargent, 2009), which is the case for *S. nutans* in Belgium, for which a specialized nursery pollinator (*Hadena albimacula*) but also other nursery pollinators (*Perizoma*, *Coleophora*) are known (De Prins and Steeman, 2021). However, pollinator assemblages of the two ecotypes have never been compared and precise knowledge on moth distribution and abundance in southern Belgium is still lacking (De Prins and Steeman, 2021). In addition, no evidence of gene flow between ecotypes could be found using molecular markers likely due to hybrid inviability (Martin et al., 2016; Martin et al., 2017), but this does not mean that there are no pollen transfers between ecotypes when populations are in close proximity. Both plant and moth species are considered rare, and sharing pollinators between ecotypes might also represent a mutual benefit (e.g. Ha et al., 2021).

In the present study, we estimated pollen dispersal and pollinator isolation (or sharing) between field populations of calcicolous and silicicolous ecotypes of *S. nutans* in southern Belgium. We used fluorescent powdered dyes as pollen analogues to quantify intra- and inter-ecotypic pollen transfers, and infrared cameras to directly observe nocturnal pollinators visiting flowers. To identify seed-eating (nursery) pollinator species, caterpillars were directly collected on *Silene* plants and reared until adult stage. Finally, seeds were collected in the studied populations and germinated to detect chlorotic hybrid seedlings resulting from inter-ecotypic crosses. In case of pollinator isolation, we expect less inter-ecotypic than intra-ecotypic pollen transfers and different pollinator communities, whereas in case of pollinator sharing, we expect no difference between intra- and inter-ecotypic pollen transfers and the occurrence of chlorotic seedlings.

## Materials and methods

### Studied species

*Silene nutans* is a diploid, insect-pollinated perennial herb species. Its wide distribution range covers Western Europe and extends to the Caucasus, southern Scandinavia and Siberia (Hepper, 1956). At the western and northern borders of its distribution, *S. nutans* is a rare species with scattered populations (Fitter, 1978; Hegi, 1979). There, it mostly occurs in xerothermophilous vegetation, such as open grasslands and forest edges on rock outcrops (Hepper, 1956; Van Rossum, 2000). Flowers open at dusk and produce nectar (Witt et al., 2013). Flower scent typically attracts nocturnal moths which are the main pollinators (Hepper, 1956; Jürgens et al., 1996; Jürgens et al., 2002). Diurnal visitors have also been observed on *S. nutans*, including honey bees (*Apis mellifera*), wild bees (e.g. *Andrena* species), bumblebees (e.g. *Bombus hortorum*, *B. terrestris*, *B. lapidarius*) and syrphid flies (e.g. *Episyrphus balteatus*) (Jürgens et al., 1996; Vanderplanck et al., 2020), but they are not effective pollinators. Indeed, pollen deposition on stigmas by diurnal visitors is rare and the resulting seed production negligible (Vanderplanck et al., 2020). Nocturnal moths pollinating *S. nutans* include Noctuidae (e.g. *Autographa gamma*), Geometridae (e.g. *Eupithecia linariata*), Crambidae (e.g. *Anania hortulata*) and Sphingidae (e.g. *Deilephila porcellus*) (Jürgens et al., 1996; Vanderplanck et al., 2020). For nursery pollinators, 11 *Hadena* species (e.g. *Hadena albimacula*, *H. bicruris*, *H. perplexa, H. filograna*), *Sideridis rivularis* (Noctuidae) and *Perizoma hydrata* (Geometridae) are known (Kephart et al., 2006; Prieto-Benítez et al., 2017; De Prins and Steeman, 2021). In particular, *H. albimacula* is only found on *S. nutans* in Belgium and Great Britain (Young, 1997; De Prins and Steeman, 2021), but it has also been reported on other *Silene* species, e.g. in southern Europe (Kephart et al., 2006; Prieto-Benítez et al., 2017; Wagner, 2020). *Coleophora* (Coleophoridae) species are also known as nursery moth pollinators of *S. nutans*, e.g. *C. albella* and *C. silenella*, the latter being probably extinct in Belgium (De Prins and Steeman, 2021).

### Studied populations

**Figure 1:**
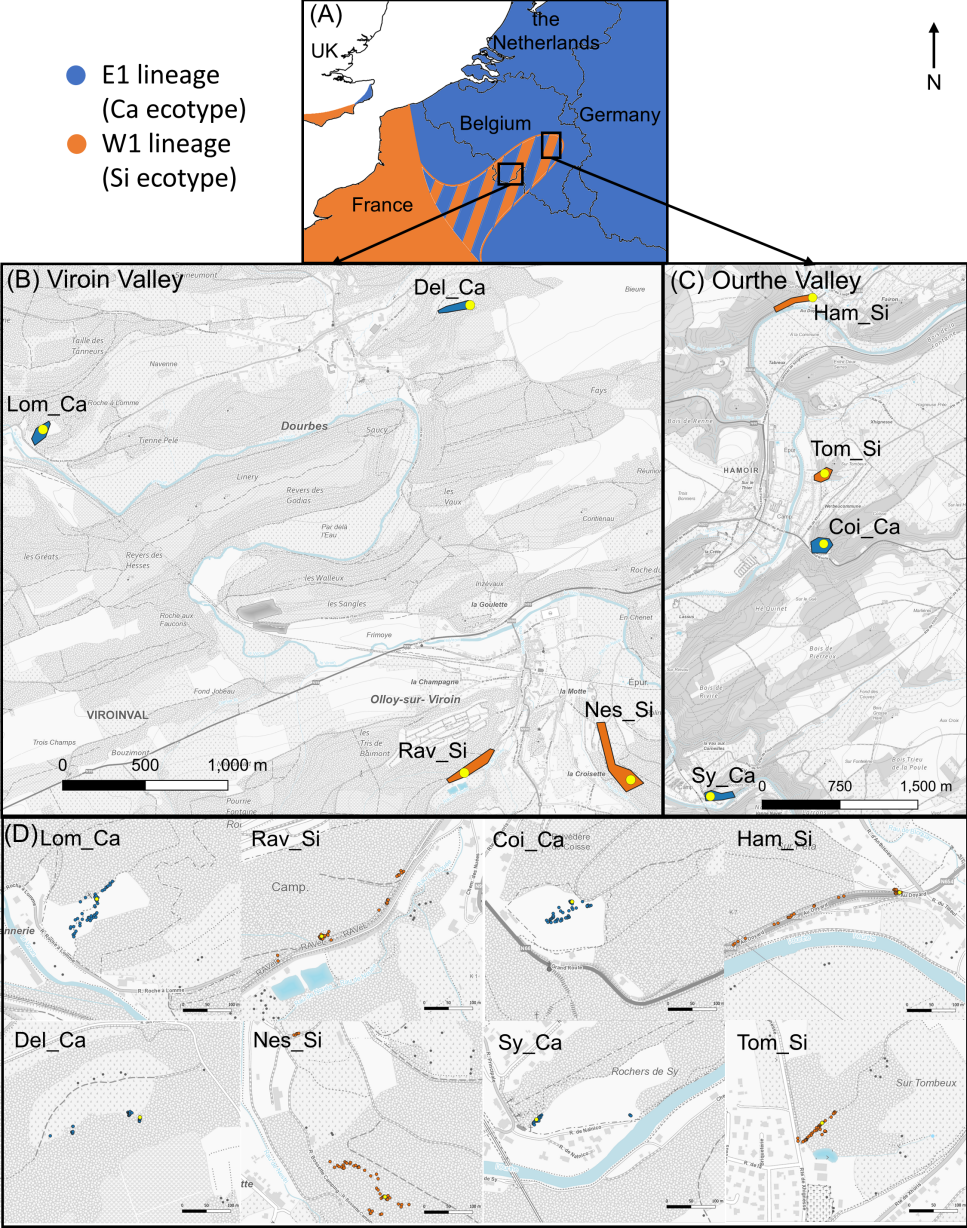
Maps showing **(A)** the general distribution of E1 and W1 genetic lineages of *Silene nutans* around the area of study, **(B, C)** the studied populations in southern Belgium (Viroin Valley and Ourthe Valley, respectively), and **(D)** the individuals studied in each sampled population. Blue and orange dots represent recipient individuals from calcicolous ecotype (E1 lineage) and silicicolous ecotype (W1 lineage), respectively. Yellow dots indicate source individuals. Source of the background maps: Institut Géographique National (2022).

The study was carried out in two regions of southern Belgium where the calcicolous (Ca) and silicicolous (Si) ecotypes of *S. nutans* occur in parapatry: the Viroin Valley and the Ourthe Valley (**Figure 1**). In each valley, four populations (two of each ecotype) were chosen so that they were separated by the shortest geographic distance (from 0.7 to 5.0 km; **Figure 1**; **Table S1**). The Ca ecotype occurred on xeric calcareous grasslands and the Si ecotype on dry acid grasslands or forest edges on schist outcrops.

### Pollen dispersal experiment using fluorescent powdered dyes

To study pollen dispersal and pollinator movements within and between populations, fluorescent powdered dyes (Radiant Color, Series Radglo^®^ R) were used as pollen analogues (Van Rossum et al., 2011). A distinct powder color was assigned to each population in each region (blue, pink, orange and yellow; **Table 1**; **Figure S1**). Field experiments were conducted in late May and early June 2019 in the Viroin Valley and in May 2020 in the Ourthe Valley, during the two weeks of overlap of the flowering periods of the ecotypes. It was not possible to conduct the experiment in the Ourthe Valley in 2019, because Sy_Ca and Coi_Ca were grazed by sheep despite agreement with the local manager for waiting to put the flock in the site. The number of flowering individuals at the time of the experiment was counted in each population (**Table 1**). In population Sy_Ca, the number of flowering individuals (20, of which 11 plants located on an unreachable cliff) was too small to conduct the experiment. Therefore, 21 additional individuals, each consisting of a few freshly harvested flowering inflorescences collected in the nearby population Coi_Ca just before starting the experiment in the evening, were placed in pots filled with water (**Figure S2**) during the time of the experiment.

**Table 1:** Population details and dye dispersal results in eight populations of *Silene nutans* from the Viroin and the Ourthe valleys in southern Belgium.

Population	*N*	*n*	S (m²)	Dye source color	Percentage of individuals with dye (%)				Percentage of flowers of recipient ind. with dye (%)			
Viroin					Del_Ca	Rav_Si	Nes_Si	Lom_Ca	Del_Ca	Rav_Si	Nes_Si	Lom_Ca
Del_Ca	45	33	6240	Blue	**75.8**	30.3	27.3	39.4	**27.9 ± 4.5**	5.8 ± 1.7	6.1 ± 1.9	9.7 ± 2.3
Rav_Si	123	41	7800	Yellow	39.0	**80.5**	22.0	39.0	9.3 ± 2.2	**53.1 ± 5.7**	6.6 ± 2.4	10.8 ± 2.5
Nes_Si	135	51	15730	Orange	54.9	49.0	**68.6**	13.7	13.3 ± 2.2	11.7 ± 2.2	**33.0 ± 4.8**	2.5 ± 0.9
Lom_Ca	300	50	4220	Pink	48.0	20.0	28.0	**56.0**	9.4 ± 1.8	2.9 ± 0.8	4.3 ± 1.0	**18.0 ± 3.6**
Ourthe					Coi_Ca	Ham_Si	Tom_Si	Sy_Ca	Coi_Ca	Ham_Si	Tom_Si	Sy_Ca
Coi_Ca	120	50	3602	Blue	**70.0**	24.0	34.0	78.0	**30.6 ± 4.8**	5.0 ± 1.5	7.1 ± 1.6	31.5 ± 4.0
Ham_Si	100	33	5109	Yellow	36.4	**90.9**	60.6	84.8	6.5 ± 1.7	**53.7 ± 6.2**	11.3 ± 1.9	39.5 ± 5.3
Tom_Si	70	40	3125	Orange	55.0	27.5	**75.0**	40.0	22.0 ± 4.6	7.9 ± 3.0	**37.7 ± 5.7**	9.4 ± 2.9
Sy_Ca	41*	26	1122	Pink	53.8	26.9	42.3	**100.0**	9.9 ± 2.4	4.0 ± 1.6	9.1 ± 2.5	**83.8 ± 3.7**

*N*, population size (number of flowering individuals); *n*, number of sampled recipient individuals; S, population area (m²); Ca, calcicolous ecotype; Si, silicicolous ecotype. Dye dispersal values are percentages of recipient individuals and flowers per recipient individual showing dye deposition (mean ± standard error), depending on dye source (columns are source populations, lines are recipient populations). Intrapopulation transfers are in bold, the other values are interpopulation data. *In population Sy_Ca, 21 individuals (inflorescences placed in pots with water) were added for the duration of the experiment.

Each hermaphrodite protandrous flower of *S. nutans* opens during a few successive nights: a first group of five stamens appears and dehisces during the first evening; the next day, it withers and is replaced by a second group of five stamens; from the third evening and for up to three days, three stigmas become receptive (Hepper, 1956; Vanderplanck et al., 2020). Powdered dyes were applied to dehiscent anthers of source individuals during two successive evenings at dusk. To ensure that moths were active, nights with no rain and suitable temperatures were chosen (minimal temperature during the three-day period of the experiment ranging 8.8-12.4°C in 2019 and 9.4-10.0°C in 2020; data from the Dourbes and Louveigné meteorological stations, respectively, provided by the Royal Meteorological Institute of Belgium). For two consecutive evenings, anthers of 62 to 77 source flowers located on a group of close individuals (approximately 0.5 m² in area) were marked in each population with a toothpick covered with dye. On the third day, 1 to 13 (usually 7) flowers were collected from 26 to 51 recipient individuals in each population (total of 170 to 350 flowers per population) in order to observe the presence of dye on stigmas. The sampled recipient individuals were representative of plant spatial distribution in the populations.

The number of inflorescences and the number of open flowers for five inflorescences were counted for each recipient individual, and used to estimate the total number of flowers (**Table S2**), which can be an indicator of plant attractiveness (Kearns and Inouye, 1993; Ghazoul, 2005). Recipient individuals were sampled across the whole population to cover a wide range of distances from source individuals (i.e. dye source) (usually 2–200 m). Each recipient individual and the source group were mapped with a GPS (Trimble^®^ GPS Pathfinder^®^ ProXRT) to calculate the distances of dye transfers (Appendix S1). The area of each population (in m²) was calculated using the vector geometry tools in QGIS (QGIS Development Team, 2020).

The three stigmas of each recipient flower were fixed with glycerin jelly (50 g gelatin in 175 ml distilled water and 150 ml glycerin; Kearns and Inouye, 1993) on a microscope slide. Slides were observed under a fluorescence microscope (Leitz Diaplan) at 250x, and the number of dye particles was counted for the three stigmas of each flower, for each dye color, and used to calculate the mean dye count per flower and the sum of dye counts for each recipient individual and dye color (for all flowers).

### Identification of visiting pollinators

To give insight into the nocturnal pollinators of *S. nutans*, observations were made with homemade infrared cameras filming continuously from dusk to dawn (**Figure S3**; Appendix S2; Droissart et al., 2021). A camera was placed in the front of the dense group of flowers chosen as source individuals for dye in each studied population (Viroin Valley: 31 May-01 June 2019; Ourthe Valley: 07 June 2019 (only for Tom_Si) and on 19-20 May 2020 (the 4 populations)). In total, 22.4 and 38.8 hours were recorded for the Ca and Si ecotypes, respectively. For each night, the number of moths appearing in the frame per hour and the number of flowers visited per hour were calculated. A flower was considered “visited” when a moth clearly touched anthers or stigmas. Mann-Whitney *U-*tests were performed in R v.3.5.1 (R Core Team, 2018) to test for differences between ecotypes in recording duration, number of flowers filmed, number of moths observed per hour and number of flowers visited per hour. Moths were identified at species, genus or family level depending on image quality.

To identify the community of nursery seed-eating pollinators, but also possibly of other seed predators of each ecotype, larvae observed on *S. nutans* flowers and capsules were noted and counted, in each population in the Viroin and Ourthe valleys during six visits in June-July 2019 and May-June 2020. In total, 25 and 200 larvae were observed in Ca and Si populations, respectively (**Table 2**). Of these, 18 and 76 larvae were collected in Ca and Si populations, respectively, in capsules, on inflorescences or in the soil near *S. nutans* (**Figures S4A, B**), for further rearing and identification at adult stage (see Appendix S3 for details). Of the reared larvae, 69 were moth caterpillars (14 in Ca and 55 in Si populations). After adult identification, seed predators of *S. nutans* were assigned to one of the five following taxonomic groups: Noctuidae (Lepidoptera, including *Hadena*), *Coleophora* (Coleophoridae, Lepidoptera), Coleoptera (two weevil species including *Hypera arator*), Diptera (likely *Delia pruinosa*) and a gall midge (*Dasineura bergrothiana*, Diptera). To identify differences in communities of seed predators between populations and ecotypes, a Principal Component Analysis (PCA) was conducted on the correlation matrix based on the abundance of each taxonomic group for each population and each year using the R package vegan (Oksanen et al., 2018). To test for differences in seed predator communities between ecotypes and years, a Permutational Multivariate Analysis of Variance (PERMANOVA) was performed using the function *adonis2* in vegan.

**Table 2:** List of the seed predators found on *Silene nutans* in eight calcicolous (_Ca) and silicicolous (_Si) populations in southern Belgium in 2019 and 2020, grouped in five taxonomic groups.

Population	Year	Lepidoptera (Noctuidae)	Lepidoptera (*Coleophora*)	Coleoptera(Curculionidae)	Diptera (*Delia*)	Diptera (gall)
Viroin						
Lom_Ca	2019	7	0	0	0	0
	2020	2	0	0	0	0
Del_Ca	2019	4	0	3	0	0
Ourthe						
Sy_Ca	2020	1	0	0	0	0
Coi_Ca	2020	6	0	0	0	0
Viroin						
Nes_Si	2019	5	2	8	25	20
	2020	3	1	2	0	0
Rav_Si	2019	6	0	7	7	1
	2020	7	1	4	0	0
Ourthe						
Tom_Si	2019	8	34	0	1	0
	2020	5	2	5	1	1
Ham_Si	2019	18	0	0	0	0
	2020	18	0	3	0	0

Noctuidae were all *Hadena* sp., with *H. albimacula* confirmed in Lom_Ca, Coi_Ca, Rav_Si, Tom_Si, and Ham_Si; in Ham_Si, *Sideridis rivularis* was also found in 2019. Coleoptera (Curculionidae) were *Hypera arator* and another unidentified weevil species. Diptera was likely *Delia pruinosa* (Anthomyiidae), and *Coleophora* was *C. albella* (Lepidoptera: Coleophoridae). The gall was *Dasineura bergrothiana* (Diptera, Cecidomyiidae).

### Dye dispersal pattern analyses

To investigate pollen flow within and between populations, dye deposition data were used (1) to determine spatial pollen dispersal patterns within and between populations; (2) to test for differences in intrapopulation dye transfer patterns among populations; (3) to compare dye transfers between populations of the same ecotype and between ecotypes to evaluate whether there was pollinator isolation or sharing (**Figure S1**); and (4) to determine if there was a preferred direction of inter-ecotypic dye transfers by pollinators (Si to Ca or Ca to Si).

To determine if there was a spatial pattern of dye dispersal, Moran’s *I* spatial autocorrelation analyses were performed on the mean dye count per flower given the inverse distance weights between recipient individuals (Bivand and Wong, 2018). We might indeed observe either a clustering of dye depositions due to nonrandom movements of pollinators or no spatial pattern if pollinators randomly flew from one plant to another within and between populations. This analysis was performed for each population (within-population dispersal) and for each dye colour (between-population dispersal) in each valley. *P*-values were obtained by permutation tests (999 permutations) using the function *moran.mc* (R package spdep, Bivand and Wong, 2018). As each analysis was repeated four times for each valley, the significance threshold was adjusted for multiple testing (Bonferroni correction; *P* = 0.0125). Spatial spline autocorrelograms of intra- and interpopulation dye transfers were computed using the function *spline.correlog* (R package ncf; Bjornstad, 2022).

To determine the function characterizing dye movement (i.e. the dye dispersal kernel) within each population, parameters *α* and *β* of the equation *f*(*α*, *β*; *r*) = [β*exp(-(*r*/*α*)*^β^*)] / [2*πα*² Γ(2/*β*)], where *r* is the distance to dye source and Γ is the Gamma function, were estimated using a chi-squared minimization in Excel Solver (Hardy et al., 2004; Van Rossum et al., 2011), based on the mean dye count per flower for each recipient individual. The parameter *α* represents the extent of dispersal while *β* represents the shape of the dispersal curve. If *β* < 1, the dispersal kernel is leptokurtic (fat-tailed), and it is thin-tailed if *β* > 1. The mean distance of dye transfer *δ_k_* was calculated with the following equation: *δ_k_* = *α*[Γ(3/*β*)/Γ(2/*β*)] (Hardy et al., 2004). Gamma (Γ) correlation coefficients were calculated between the mean dye count per flower and the distance to dye source for each population using STATISTICA version 12 (Dell Inc.).

To test for differences in dye transfers between populations in each valley, we used hurdle models fitted to the sum of dye counts (Appendix S4) to perform three analyses that aimed: (1) to test for differences in intrapopulation dye transfers between populations; (2) to test for differences in dye transfers between populations of the same ecotype and of different ecotypes; and (3) to determine whether there was a directionality of pollen flow (from Si to Ca or from Ca to Si), with the dataset restricted to inter-ecotypic transfers. For both parts of the hurdle model, the distance to dye source and the total number of flowers per plant were used as quantitative explanatory variables, while the type of dye transfer (intra-ecotype *vs.* inter-ecotype and from Si to Ca *vs.* Ca to Si for analyses 2 and 3, respectively) or population for analysis 1 as nominal explanatory variable. The interaction between distance to dye source and type of transfer (analyses 2 and 3) or population (analysis 1) was also included in the analyses. The number of sampled flowers per recipient individual was used as an offset (Zuur et al., 2009). The best fitting parameters were estimated using a maximum likelihood approach and the significance of the coefficient estimates was calculated using a Wald test (α = 0.05). Analyses were performed using the *hurdle* function included in the R package pscl (Zeileis et al., 2008; Jackman, 2020) and the *lrtest* function in the lmtest package (Zeileis and Hothorn, 2002). The absence of spatial autocorrelations in the residuals, assumed by the model, was verified using the *testSpatialAutocorrelation* function (based on Moran’s *I* test) from the R package DHARMa (Hartig, 2022).

### Seed germination experiment

To detect hybrids from inter-ecotypic crosses, seeds were collected across the eight populations in June 2019 and/or 2020 and stored in a dry-cold environment (15°C, 15% relative humidity). A bulk of 100-200 seeds per population were germinated in 4-8 Petri dishes on 1% agar (10 g/l) in an incubator at 20°C and 8/16 (light/dark) photoperiods. The percentage of germinated seeds was recorded after two weeks. Chlorotic (yellow-white) or partially chlorotic (light green) seedlings likely corresponding to inter-ecotype hybrids (Martin et al., 2017; **Figure S5**) were counted. Goodness of fit (Chi square) tests comparing seed germination and proportion of chlorotic seedlings between ecotypes were performed using the function *chisq.test* in R.

## Results

### Identification of pollinators and seed predators

**Figure 2:**
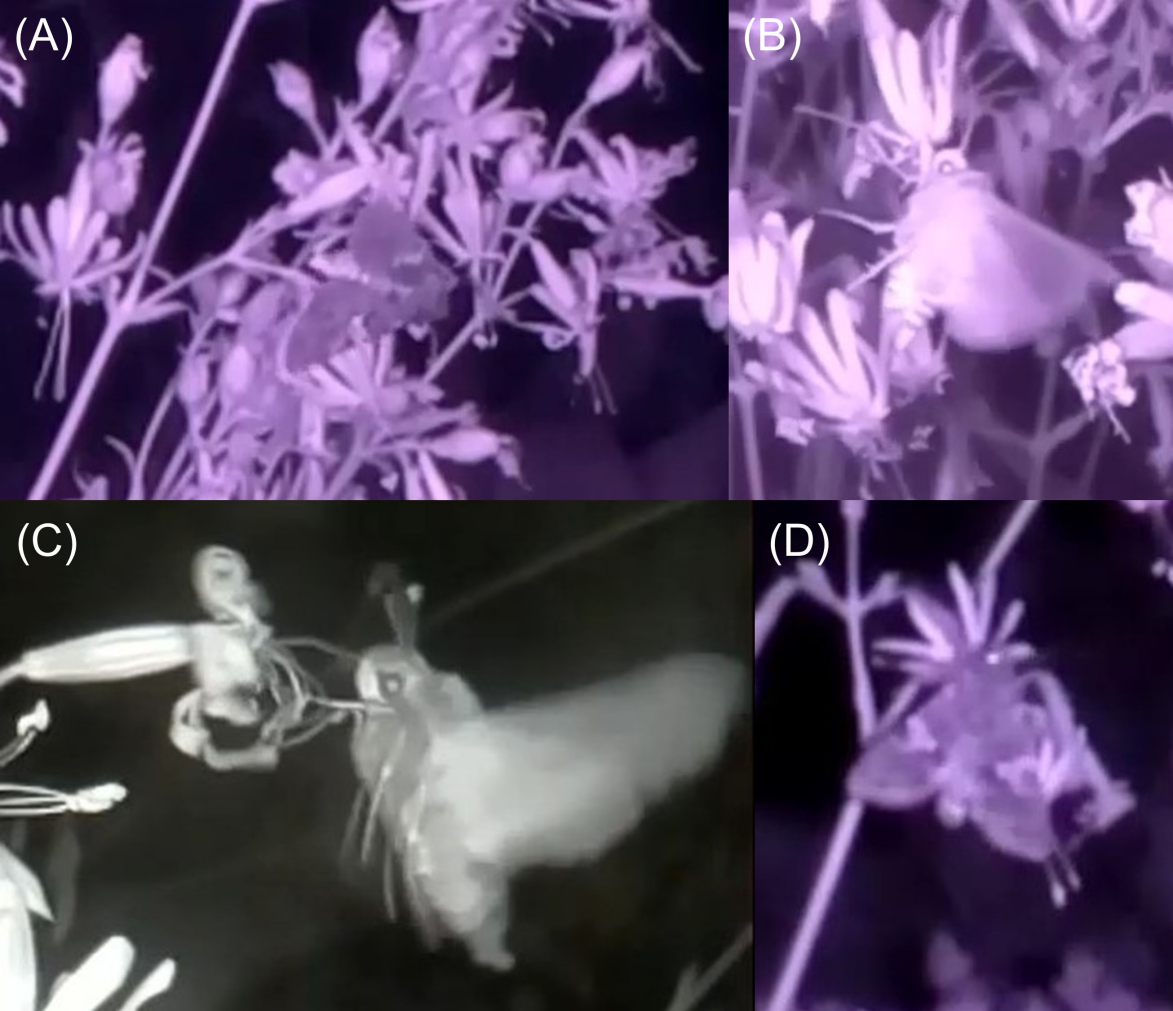
Examples of nocturnal pollinators of *Silene nutans* observed with homemade infrared cameras in 2019 and 2020: **(A)** *Hadena* sp., **(B)** Noctuidae, **(C)** *Proserpinus proserpina*, and **(D)** *Hadena albimacula*.

The total number of moths recorded with the cameras was 33 and 11 in Si and Ca populations, respectively (**Table 3**). Moths were often moving too fast on the images for unequivocal identification at species level, but could mostly be identified as Noctuidae. Geometridae and Sphingidae were also observed (**Table 3**). *Hadena albimacula* visited both Si and Ca flowers, and was filmed laying eggs in population Sy_Ca (**Figure 2**; Cornet et al., 2020). No significant difference was found between ecotypes in the number of filmed flowers, mean recording duration, and in the number of moths and visited flowers per hour (Mann-Whitney *U*-tests ≤ 29.5, *P* > 0.200; **Table S3**).

**Table 3:** Results of infrared cameras filming nocturnal pollinators in eight calcicolous (Ca) and silicicolous (Si) populations of *Silene nutans* from the Viroin Valley in 2019 and the Ourthe Valley in 2019 and 2020.

Valley	Population	Date	Recording duration (h)	Number of flowers filmed	Number of flowers visited	Number of moths observed	Number of flowers visited per h	Number of moths observed per h	Taxon
Viroin	Del_Ca	01-06-19	7.25	34	1	1	0.14	0.14	Geometridae
	Lom_Ca	31-05-19	2.17	40	1	1	0.46	0.46	Microlepidoptera
Ourthe	Coi_Ca	19-05-20	2.83	78	0	0	0	0	
		20-05-20	2.83	58	4	2	1.41	0.71	*Hadena* sp.; Noctuidae
	Sy_Ca	19-05-20	2.75	51	0	0	0	0	
		20-05-20	4.58	85	16	7	3.49	1.53	*Hadena albimacula;* Sphingidae; Noctuidae
Viroin	Nes_Si	31-05-19	7.67	28	0	1	0	0.13	Noctuidae
	Rav_Si	01-06-19	7.5	15	12	7	1.6	0.93	*Proserpinus proserpina*; Geometridae; Noctuidae
Ourthe	Ham_Si	19-05-20	5.83	73	20	10	3.43	1.72	*Hadena albimacula;* Noctuidae
		20-05-20	2.67	35	10	6	3.75	2.25	*Hadena albimacula*; Noctuidae
	Tom_Si	07-06-19	7.08	27	11	6	1.55	0.85	*Autographa gamma*; Noctuidae
		19-05-20	2.75	69	0	0	0	0	
		20-05-20	5.25	55	18	3	3.43	0.57	*Hadena albimacula*; Noctuidae

**Figure 3:**
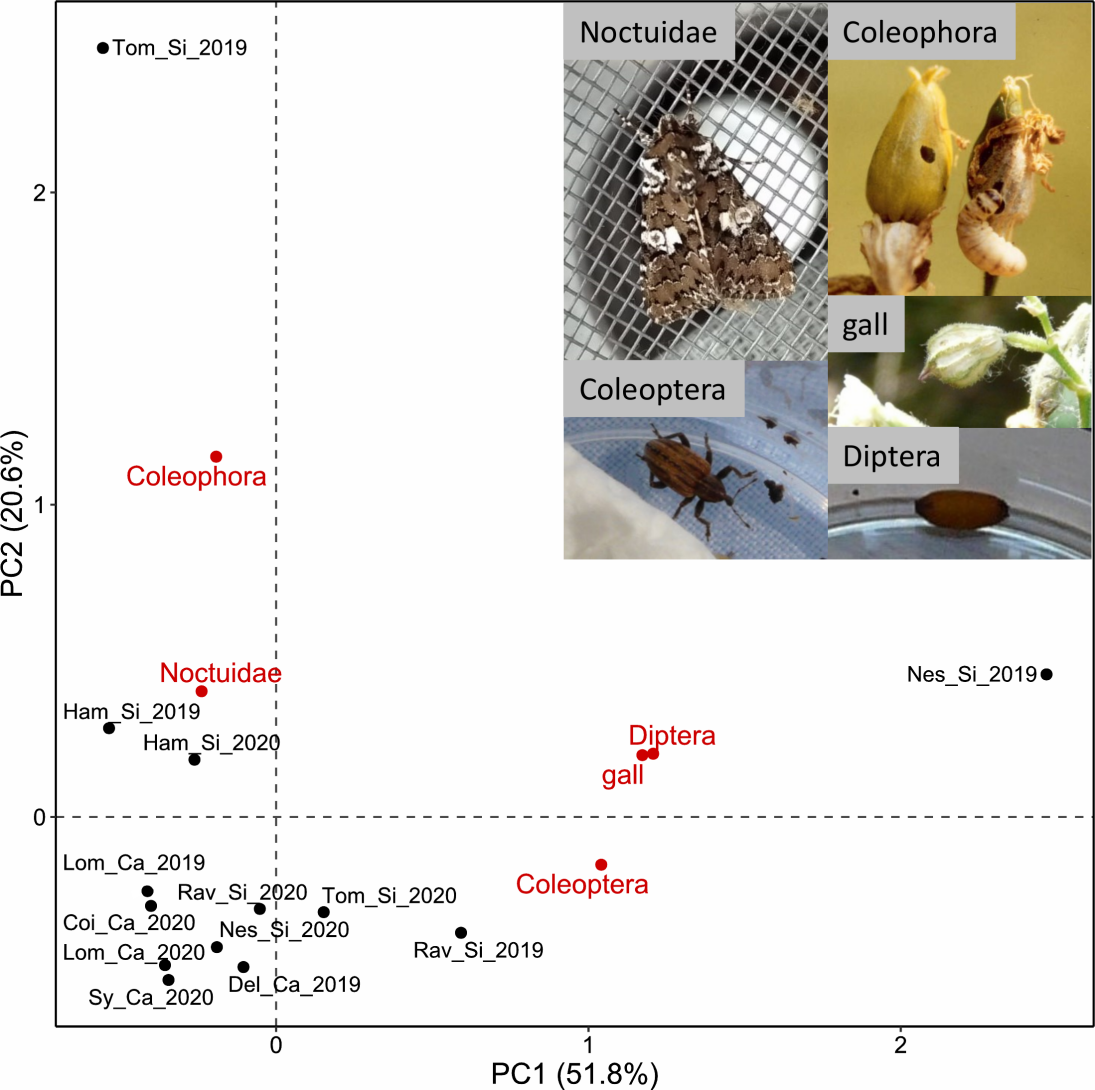
Principal Component Analysis on the number of seed predators of *Silene nutans* found in eight calcicolous (Ca) and silicicolous (Si) populations (black dots) from southern Belgium in 2019 (_19) and 2020 (_20), grouped in five taxonomic groups (Noctuidae, *Coleophora*, Coleoptera (Curculionidae), Diptera (*Delia*) and gall; see **Table 2**) and shown as centroids (red dots).

In total, 70 Noctuidae, 40 *Coleophora*, 29 Coleoptera (Curculionidae), 34 Diptera (*Delia*) and 22 galls (*Dasineura bergrothiana*, Diptera) were observed on Si plants, and 20 Noctuidae and three Coleoptera on Ca plants (**Table 2**). One additional species of Noctuidae was observed: *Sideridis rivularis*, of which an empty pupal case was found in Ham_Si. Rearing of collected seed-predator larvae led to 33% of individuals dying before reaching pupal stage, 35% dying during pupal stage and 32% having completed development until adult stage, for a total of 57 reared larvae. No difference in survival rates was observed between ecotypes (Fisher’s exact test, *P* = 0.753). All moth caterpillars having reached adult stage were identified as *Hadena albimacula* (**Figure S4E**), of which 11 individuals were found in Si populations and two in Ca populations. The first two axes of PCA on the abundance matrix of seed predators grouped as five taxonomic groups (**Figure 3**) explained 72.4% of the total variance. The first axis was related to higher number of Diptera (correlation coefficient *r* = 0.97), and the second axis to the presence of *Coleophora* (*r* = 0.93), allowing to distinguish Ca from most Si populations where more predators were observed. *Coleophora*, certainly *Coleophora albella*, the Coleoptera (two species of weevils), the Diptera and the gall were only found in Si populations, except for the weevil *Hypera arator* also found in Del_Ca (**Table 2**). Differences between ecotypes significantly explained the variation in the dataset (PERMANOVA, *F* = 3.15, *P* = 0.005), while year of data collection had no significant effect (*F* = 1.05, *P* = 0.404). Two populations were clearly distinct from the others: Nes_Si in 2019, where numerous Diptera larvae and galls were observed, and Tom_Si in 2019 where many caterpillars of *Coleophora albella* were found. The highest number of Noctuidae caterpillars was found in Ham_Si. Calcicolous populations appeared less different from each other than Si populations (**Figure 3**), probably due to the absence of most taxonomic groups except Noctuidae in this ecotype (**Table 2**).

### Dye dispersal patterns

**Figure 4:**
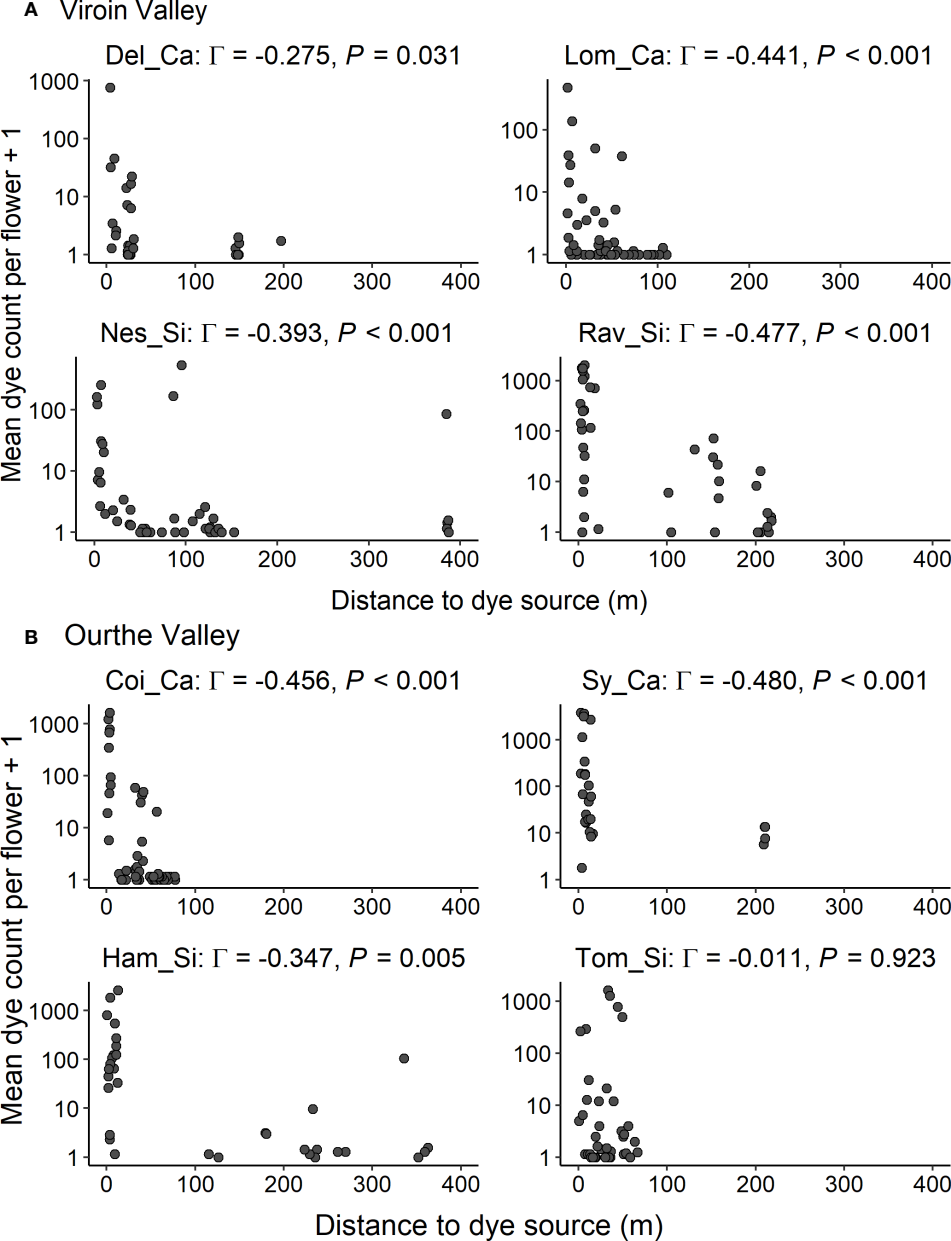
Intrapopulation dye transfers: mean dye count per flower for each recipient individual (axis in log-scale) in function of the distance to dye source for eight calcicolous (Ca) and silicicolous (Si) populations of *Silene nutans* in **(A)** Viroin Valley and **(B)** Ourthe Valley. Γ: Gamma correlation coefficient.

**Figure 5:**
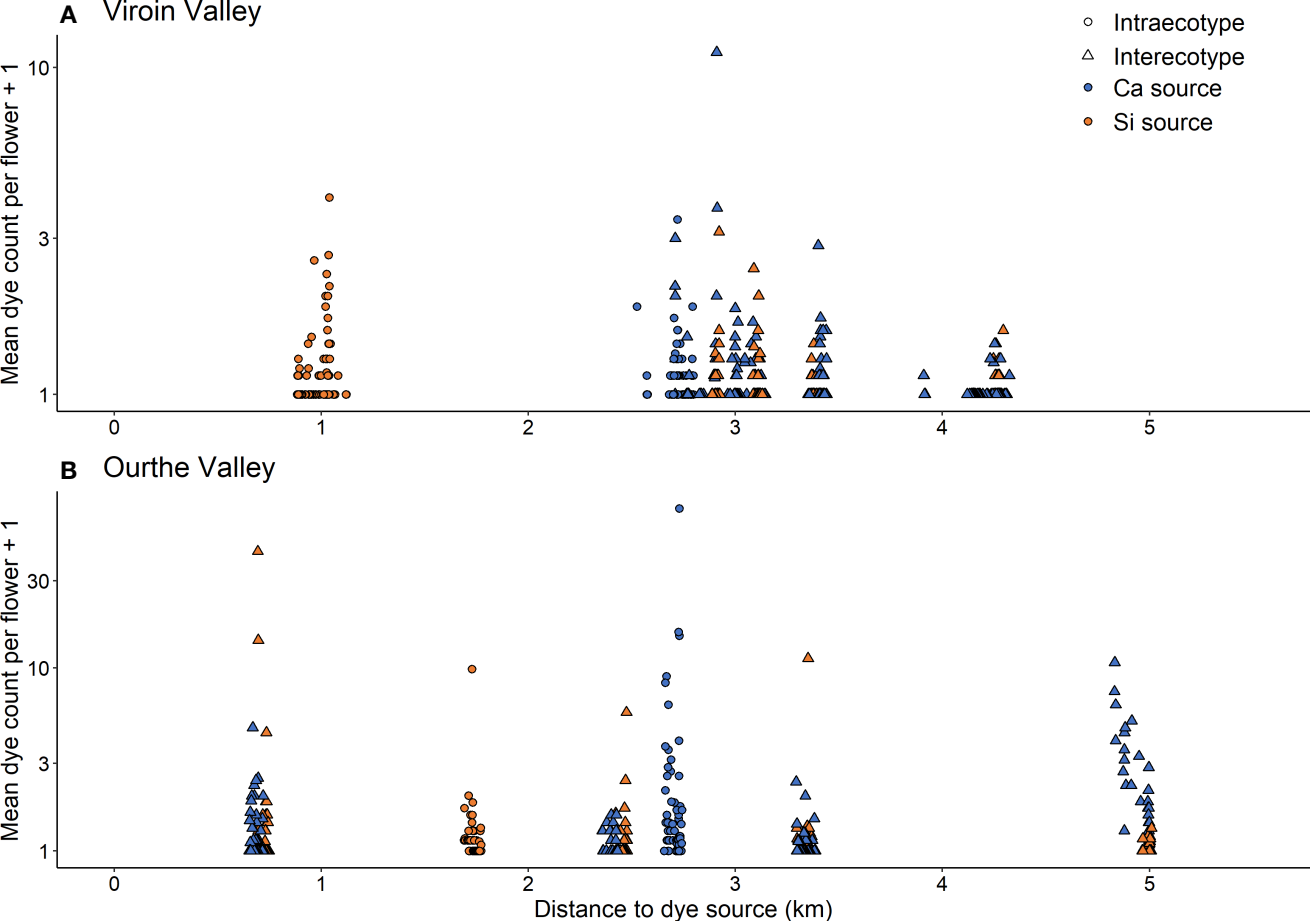
Mean dye count per flower (axis in log-scale) or each recipient individual and dye source in function of the distance to dye source for interpopulation dye transfers between four calcicolous (Ca) and silicicolous (Si) populations of *Silene nutans* populations in **(A)** Viroin Valley and **(B)** Ourthe Valley. Circles = intra-ecotypic transfers (blue, Ca; orange, Si); triangles = inter-ecotypic dye transfers (blue: Ca to Si; orange: Si to Ca).

In both regions, dye transfers were observed within all populations and between all dye source and recipient populations. The percentage of individuals showing dye deposition ranged from 56.0% to 100.0% for intrapopulation transfers, from 22.0% to 78.0% for interpopulation intra-ecotypic transfers and from 13.7% to 84.8% for interpopulation inter-ecotypic transfers, with a range of mean percentage of flowers of recipient individuals showing dye of 18.0-83.8%, 6.6-31.5% and 2.5-39.5%, respectively (**Table 1**). Dye transfers within populations reached up to 386 m (**Figure 4**), and there were interpopulation dye transfers up to 4.32 km (Viroin Valley) and 5.01 km (Ourthe Valley) (**Figure 5**).

#### Within-population dye dispersal

In the Viroin Valley, dye deposition within populations was random (i.e. no spatial pattern), for all four populations (Moran’s *I* spatial autocorrelation statistics not significant, ranging from -0.01 to 0.10, *P* > 0.050; **Figure S6A**). In the Ourthe Valley, significant spatial clustering of dye deposition was observed in Coi_Ca (Moran’s *I* = 0.52, *P* = 0.001) and Sy_Ca (*I* = 0.23, *P* = 0.007), but not in Tom_Si and Ham_Si (*I* = 0.05 and -0.04, respectively, *P* > 0.050; **Figure S6B**). The shape of the dye dispersal distribution was leptokurtic (*β* < 1), except for Nes_Si and Ham_Si, for which the distribution was more linear (*β* > 1) (**Figure 4**; **Table 4**). The mean distance of dye transfer (*δ_k_*) ranged from 18.1 m in Del_Ca to 2,642 m in Lom_Ca. For Tom_Si, no best fitting values for the parameters *α* and *β* of the dye dispersal kernel could be calculated, and very low best fitting values were obtained by Solver for *α* for Del_Ca and Lom_Ca, so that their *δ_k_* values might be questioned. This was likely because the dispersal could be considered as random as indicated by the non-significant Moran’s *I* statistics (**Table 4**). All other populations showed a significant decrease of mean dye count with distance to dye source (Gamma correlation coefficients ranging from -0.270 to -0.480, *P* ≤ 0.030).

**Table 4:** Results of dye dispersal distribution patterns within eight calcicolous (Ca) and silicicolous (Si) populations of *Silene nutans* from the Viroin and the Ourthe valleys: Moran’s *I* spatial autocorrelation statistics, best fitting *α* (extent of dye dispersal) and *β* (shape of dye dispersal curve), *δ_k_* (mean distance of dye transfer), and Gamma correlation coefficient (Γ).

Population	Moran’s *I*	*α*	*β*	*δ_k_* (m)	Gamma correlation	
					Γ	*P* value
Viroin						
Del_Ca	0.01ns	0.0001	0.20	18.1	-0.275	0.031
Rav_Si	0.10ns	28.9	0.83	87.7	-0.477	< 0.001
Nes_Si	-0.01ns	388.0	2.98	287.0	-0.393	< 0.001
Lom_Ca	-0.01ns	0.0001	0.16	2,642.0	-0.441	< 0.001
Ourthe						
Coi_Ca	0.52*	5.1	0.73	21.9	-0.456	< 0.001
Ham_Si	-0.04ns	99.3	1.08	173.0	-0.347	0.005
Tom_Si	0.05ns	–	–	–	-0.011	0.923
Sy_Ca	0.23*	1.8	0.39	160.0	-0.480	< 0.001

ns, not significant (*P* > 0.05); *, significant (*P* < 0.05).

The hurdle model applied on intrapopulation transfers revealed differences among populations (**Table S4**). In particular, the probability of dye transfer was significantly higher for Rav_Si population than for the other populations of the Viroin Valley (Wald test, *Z* = 2.830, *P* = 0.005). Overall, a significant decrease in dye transfers with distance to dye source was observed, except for the Viroin Valley where the distance did not affect the dye count in case of dye transfer (but the probability of transfer decreased with distance). In the Ourthe Valley, distance to dye source differently affected the probability of dye transfer depending on population (significant interactions between distance to dye source and population, *P* ≤ 0.001).

#### Among-population dye dispersal

In the Viroin Valley, dye deposition among populations showed significant spatial clustering for Lom_Ca and Rav_Si source populations (Moran’s *I* = 0.19, *P* = 0.003 and *I* = 0.34, *P* = 0.001, respectively; **Figure S7A**). In the Ourthe Valley, dye deposition showed significant spatial clustering only for Sy_Ca as source (*I* = 0.14, *P* = 0.003; **Figure S7B**).The spatial autocorrelation analysis was not significant for the other source populations (*I* ranging from -0.04 to 0.09, *P* > 0.050; **Figure S7**).

The hurdle models comparing intra- *vs.* inter-ecotypic interpopulation transfers showed contrasted results (**Table S5**). In the Viroin Valley, one individual of Rav_Si population showed an extremely high dye count from Del_Ca (**Figure S8A**) and was considered as an outlier. As this outlier alone substantially changed the outcome of the hurdle model (significantly higher dye count for inter-ecotypic transfers), it was removed from the analysis. The probability of dye transfer in the Viroin Valley was higher in case of inter-ecotypic than in case of intra-ecotypic transfers (*Z* = 2.65, *P* = 0.008). There was a significant interaction between type of transfer and distance to dye source (*Z* = -2.94, *P* = 0.003), i.e. the probability of dye transfer decreased with distance to dye source for inter-ecotypic transfers while it increased with distance for intra-ecotypic transfers. As the range of distance to dye source was shorter for intra-ecotypic transfers (0.8 to 2.8 km) than for inter-ecotypic transfers (2.7 to 4.3 km, **Figures 1**, **5A**) in the Viroin Valley, we performed new analyses on distances ranging from 2.5 to 3.5 km. When considering similar distance ranges (“type of transfer” not significant anymore), there was no actual difference between intra- and inter-ecotypic transfers. Spatial autocorrelations in the residuals were not significant for the Viroin Valley (Moran’s *I* = 0.045; *P* = 0.303) and the Ourthe Valley (Moran’s *I* = 0.060; *P* = 0.206), indicating that the assumption of absence of spatial autocorrelation was respected and that dye dispersal patterns were not related to plant spatial distribution.

In the Ourthe Valley (**Figures 5** **B**; **Figure S8B**; **Table S5**), results were different. The probability of dye transfer (presence/absence) and the dye count in case of transfer both increased with the distance to dye source (*Z* = 2.88, *P* = 0.004 and *Z* = 2.43, *P* = 0.015, respectively). The increases were higher for intra-ecotypic transfers than for inter-ecotypic transfers (significant interaction between distance and type of transfer; *Z* = -2.45, *P* = 0.014 and *Z* = -2.62, *P* = 0.009, for the two parts of the hurdle model, respectively; **Table S5**). Neither the total number of flowers per plant nor the type of transfer influenced the probability of dye transfer, but the dye count in case of transfer slightly increased with the total number of flowers per plant (*Z* = 2.15, *P* = 0.031; **Figure S9**), and in case of inter-ecotypic compared to intra-ecotypic transfers (*Z* = 2.90, *P* = 0.004). This meant that inter-ecotypic dye transfers were as likely as intra-ecotypic, but that the amount of transferred dye was larger for inter-ecotypic than for intra-ecotypic transfers.

When investigating the directionality of dye transfers, we found a significantly higher probability of dye transfer from Ca to Si than from Si to Ca populations in the Viroin Valley (*Z* = 2.67, *P* = 0.008; **Table S6**). The probability of dye transfer decreased more markedly with distance to dye source for Ca-Si than for Si-Ca (significant interaction between distance and type of transfer, *Z* = -2.34, *P* = 0.019). No significant effect of any variable was observed in this valley, neither on the probability of transfer nor on the dye count in case of transfer (*Z* ranging from -1.31 to 1.47, *P* > 0.050; **Table S6**).

### Germination experiment

Germination rates varied from 63 to 98% (**Table S7**) and were higher for Si populations than Ca populations (χ² = 20.51, *P* < 0.001). Chlorotic or partially chlorotic seedlings (hybrids) that indicated inter-ecotypic pollination events were found in three Si populations and in three Ca populations from both valleys, however at very low percentages (ranging from 1.1 to 4.1%) (**Table S7**; **Figure S4**). Ca and Si ecotypes did not significantly differ in percentages of chlorotic seedlings (χ² = 1.60, *P* = 0.206).

## Discussion

In this study, we investigated pollen dispersal between and within populations using fluorescent powdered dyes, pollinator and seed predator communities and hybrid occurrence in seeds of two reproductively isolated ecotypes of *S. nutans* which occur in parapatry in two valleys of southern Belgium. Long-distance dye transfers and very few differences in pollinator assemblages between ecotypes as well as hybrid (chlorotic) seedlings were detected, demonstrating pollinator sharing. However, seed-eating predators were more diverse in silicicolous populations than in calcicolous populations.

### Shared pollinators and different seed predator communities

Based on camera observations, the most abundant moths visiting the two ecotypes were Noctuidae, but Geometridae and Sphingidae were also observed (**Table 3** and **Figure 2**), which is consistent with previous studies on *S. nutans* pollination (Jürgens et al., 1996; Vanderplanck et al., 2020). *Hadena albimacula*, the nursery pollinator specialist on *S. nutans* in southern Belgium (Young, 1997; De Prins and Steeman, 2021), visited flowers of both ecotypes. This is confirmed when examining caterpillar rearing: *H. albimacula* appeared to be the main nursery pollinator species on both ecotypes. Other *Hadena* species, such as *H. perplexa* and *H. filograna*, are known as nursery pollinators of *S. nutans*, but they are almost extinct in Belgium (De Prins and Steeman, 2021). Except for the nursery pollinator *Coleophora albella* (and one pupa of *Sideridis rivularis*), only found in silicicolous populations, no difference in pollinator assemblages could be highlighted between ecotypes (**Table 3**), suggesting a large overlap in their pollinators. However, identification was often not possible to species level, and a low number of caterpillars reached adult stage, which do not allow us to precisely estimate the extent of overlap between pollinator assemblages. In addition, pollinator distribution and abundance are known to greatly vary between years (Kearns et al., 1998; Kephart et al., 2006; Hahn and Brühl, 2016), and data only spanned over two years.

While ecotypes of *S. nutans* did not differ in terms of pollinator identity, Noctuidae caterpillars and the other seed predators were more numerous or only found (*Coleophora* and *Delia* species) in silicicolous populations (**Table 2**), even though population sizes were similar between ecotypes. A first explanation for such a higher or specific predation pressure in silicicolous populations might be related to flower and fruit size. Flowers, capsules and seeds of the silicicolous ecotype are 1.2, 1.4 and 1.8 times larger or bigger, respectively, than those of the calcicolous ecotype (De Bilde, 1973; Van Rossum et al., 1996). Thus, the silicicolous ecotype might be more attractive, not only to pollinators in general but also to nursery pollinators and other seed predators, in particular those with larvae remaining inside the capsules (e.g. *Coleophora* and *Delia* species; Ellis, 2020; De Prins and Steeman, 2021). On the other side, capsules of the calcicolous ecotype may be too small for them. More research is needed to determine if nursery pollinators and seed predators prefer laying eggs on silicicolous plants compared to calcicolous plants, e.g. by oviposition choice experiments, and to understand the floral traits that could be involved in this preference (e.g. flower or ovary size or shape, and floral scent; Biere and Honders, 2006; Page et al., 2014; Prieto-Benítez et al., 2017). However, it is also possible that females laid as many eggs on both ecotypes, but that larval development and survival are lower on calcicolous plants, due to lower host plant quality (Gols et al., 2008). Second, calcicolous populations are located in natural reserves managed by sheep grazing (calcareous grassland conservation). Sheep graze on *S. nutans* inflorescences, eating deposited eggs/larvae and ultimately leaving less capsules for seed predators. Given that large caterpillars take shelter under the plants, we may also wonder whether soil trampling by sheep could affect caterpillar survival. This might account for the greater seed predator diversity in silicicolous populations (Kearns et al., 1998; Littlewood, 2008). A third possible explanation is that the W1 genetic lineage of *S. nutans* (i.e. silicicolous ecotype in Belgium), might have been followed by its specialized seed predators during postglacial recolonization of Europe. Such a pattern was recently detected for *Microbotryum* fungi, its genetic structure mirroring that of its host plant *S. nutans* (Hartmann et al., 2020). The two ecotypes might also vary in defensive secondary metabolites, preventing seed predator shift from silicicolous to calcicolous populations (Gols et al., 2008).

Some insect communities rely on floral and fruit production of *S. nutans* for their reproduction. Thus, conservation of *S. nutans* populations is important, not only for the plant species, but also for all associated pollinators and predators. In our study, nine insect species associated to *S. nutans* have been recorded (**Tables 2** and **3**), including rare species like *Hadena* and *Coleophora* nursery pollinators (De Prins and Steeman, 2021), but also *Delia pruinosa* and the gall midge *Dasineura bergrothiana* (Roskam and Carbonnelle, 2015; Ellis, 2020). Therefore, the two ecotypes of *S. nutans* in southern Belgium might need different conservation strategies according to distinct habitat and insect-community specificities. In protected calcicolous populations, management might be adapted to increase nursery pollinators (e.g., by delaying sheep grazing or excluding a part of the population from grazing). Protection of the silicicolous populations should be considered, and attention should be paid to prevent encroachment. Moreover, some silicicolous populations might be of conservation priority due to the presence of very rare insect species, e.g. Tom_Si and Nes_Si for the conservation of *Coleophora albella* and *Dasineura bergrothiana*. However, we only have two years of records of seed predation, and as the distribution and abundance of seed predators may greatly vary between years (Kephart et al., 2006), observations should be repeated on the long term to get a better view of seed predator population dynamics.

### Long-distance and random dye dispersal patterns within and between populations and ecotypes

Nocturnal moths, especially Noctuidae, the main pollinators of *S. nutans*, are known to be very mobile, and able to fly over several kilometers the same night (Jones et al., 2016), crossing long distances between floral visits. Moths indeed forage for nectar, but also seek for a mate or a host to lay eggs, in particular nursery pollinators (Young, 1997; MacGregor et al., 2015). These long-distance flights lead to long-distance pollen dispersal between populations (Kwak et al., 1998; Ghazoul, 2005; Barthelmess et al., 2006).

Although the fluorescent powdered dye method does not allow to distinguish between primary and secondary dye dispersal (i.e. dye particles deposited by a first pollinator that are picked up again by another pollinator and deposited on another flower; Inouye et al., 1994), the dye dispersal patterns we observed are consistent with moth flight abilities, allowing for long-distance interpopulation pollen transfers. Indeed, dye transfers that mimicked pollen dispersal were observed across populations within all studied *S. nutans* populations, with some of the most distant individuals (up to 386 m, the longest intrapopulation distance considered) having received large amounts of dye particles (**Figure 4**). Numerous long-distance dye dispersal events were also observed among populations within each region, over distances up to 5 km (the longest distance considered in this study; **Figure 5**). The lack of spatial clustering of dye deposition for most intra- and interpopulation transfers suggests that *S. nutans* pollinators fly randomly within and between populations (**Figures S6, S7**), with distant individuals and populations having a high probability to be visited and receiving pollen.

When bees and bumblebees are the main pollinators, dye dispersal patterns showed very important dye depositions close to the dye source, followed by a sharp decrease in dye transfers as the distance to source increased, i.e. a highly leptokurtic distribution, reflecting bee foraging behavior. Indeed, bees usually move from one flower or plant to the next nearby flowers or individuals, which results in a majority of pollen or dye deposition at short distances from the source donor (Kwak et al., 1998; Van Rossum et al., 2011; Mayer et al., 2012). Moths are generally considered highly mobile pollinators (Wessinger, 2021). Studies using genetic markers have found that moths dispersed pollen farther than bees, both within and between populations, and that moth pollination promotes gene flow and outcrossing (Brunet and Sweet, 2006; Rhodes et al., 2017; Skogen et al., 2019). In particular, for *Silene alba*, which is pollinated by moths and bees, moths moved dyes much farther than bees (Young, 2002), and contributed more to interpopulation gene flow (Barthelmess et al., 2006). The randomness of pollen movement in moth pollination compared to bee pollination might be explained by differences in the biology of these pollinator groups. For example, moths do not only forage for food, but also search for a mate or an oviposition site, they do not return to a colony or a nesting site, and they heavily rely on olfactory cues, as well as pheromones for mating as floral scent for finding flowers (Young, 1997; Kwak et al., 1998; Jürgens et al., 2002; Ghazoul, 2005; Kinoshita et al., 2017). In our study system with nursery pollinators, females lay a single egg per flower (Kephart et al., 2006; this study: Cornet et al., 2020) and scatter eggs between different host plants to avoid competition and cannibalism among larvae (Young, 1997), which may lead to increasing flight distances among visited flowers. Egg laying behavior might thus have contributed to the observed distant pollen transfers by *Hadena* females in *S. nutans*. However, for *Hadena bicruris*, no difference in dispersal patterns has been found between male and female moths, which are both efficient pollinators of *Silene latifolia* (Labouche and Bernasconi, 2010). More research is certainly needed to better understand which factors (e.g., floral scent, pheromones, flower abundance, landscape structure) influence nursery moth pollinator flight behavior within and among their host plant populations (Kephart et al., 2006; Hossaert-McKey et al., 2010; Hahn and Brühl, 2016).

There was evidence of directionality of dye transfers in the Viroin Valley, from calcicolous to silicicolous populations, with particularly efficient dye dispersal from the largest Lom_Ca population as source to the other populations (**Table S6**). However, no preferred direction of dye transfers was detected in the Ourthe Valley (**Table S6**). As Lom_Ca had the largest flowering size of the studied populations (**Table 1**), we might expect that this population would be more attractive to pollinators than the others, resulting in higher intrapopulation plant visitation rates and dye transfers, and less pollinator movements to smaller populations (Kwak et al., 1998; Van Rossum and Triest, 2010), which is not what we observed. In addition, it is unclear how floral density and population size might affect moth foraging behavior (Elzinga et al., 2005; Ghazoul, 2005). Another explanation might be that the experiment was conducted later in the flowering period of the calcicolous ecotype in the Viroin Valley than in the Ourthe Valley. As caterpillars preferably feed on immature capsules (Prieto-Benítez et al., 2017), the *Hadena* nursery pollinators might leave the end-flowering calcicolous populations once visited for searching for more attractive populations for reproduction and egg laying, i.e. silicicolous populations that had started to flower. To test if the silicicolous ecotype might be preferred by nursery pollinators, choice experiments could be conducted (e.g. Pascarella, 2007). We also need to better understand which floral traits play a role in the attractiveness of *S. nutans* flowers to (nursery) pollinators, e.g., flower color (Page et al., 2014) and floral scent (Waelti et al., 2008), and how the scent changes with capsule formation (Hossaert-McKey et al., 2010). In addition, the regions differ in the spatial distribution of the populations and were not investigated the same year. Pollinator populations might vary from year to year, depending on factors such as flowering abundance, pollinator abundance, climate conditions and site management (Kearns et al., 1998; Kephart et al., 2006; Hahn and Brühl, 2016). This might also explain the observed differences between regions.

Dye transfers occurred between all studied populations of *S. nutans* in each region (**Figure 5**), suggesting that populations are still connected by contemporary gene flow, despite habitat fragmentation. Efficient gene flow between populations of the same ecotype in southern Belgium was already suggested based on allozyme genetic variation (Van Rossum et al., 1997). However, no gene flow between ecotypes was found based on molecular markers (Van Rossum et al., 1997; Martin et al., 2016), whereas we found numerous inter-ecotypic dye transfers and chlorotic (hybrid) seedlings, indicating successful inter-ecotypic pollination and fertilization events (Martin et al., 2017). Therefore, populations of the two ecotypes of *S. nutans* do share pollinators and exchange pollen. Pollinator isolation does not constitute a prezygotic barrier to reproduction between the two ecotypes, but post-pollination barriers to reproduction, especially plastid-nuclear incompatibilities resulting in inviable chlorotic hybrids, obviously keep the two ecotypes as distinct evolutionary units (Martin et al., 2017; Postel et al., 2022).

### No pollinator isolation but pollinator sharing

Pollinator isolation can be expected between genetically distinct lineages due to mating costs associated with interlineage pollination events (pollen losses and investment in inviable hybrids), and the incompleteness of other prezygotic barriers (e.g., partial phenological isolation, and habitat isolation but no geographic isolation) (Baack et al., 2015; Moreira-Hernández and Muchhala, 2019; Ramírez-Aguirre et al., 2019). The two ecotypes of *S. nutans* might thus benefit from attracting different pollinator assemblages, but we found no pollinator isolation. Several hypotheses might explain our findings.

First, despite there was indication of a possible specific nursery pollinator (*Coleophora*) for the silicicolous ecotype, this moth may be too rare for favoring pollinator specialization and isolation (Coyne and Orr, 2004; Kay and Sargent, 2009). Additionally, phenological isolation (however incomplete) between ecotypes (De Bilde, 1973), might effectively reduce inter-ecotypic pollen flow and associated mating costs (Coyne and Orr, 2004). Selective pressure for a divergence in pollinator assemblages between ecotypes might therefore be weak, particularly given that *S. nutans* is a perennial plant species (Hepper, 1956), and given the edaphic specialization of the two ecotypes (De Bilde, 1977; De Bilde and Lefèbvre, 1990; Van Rossum et al., 1999), which might lead to hybrids maladapted to parent habitat conditions (Melo et al., 2014; Cahenzli et al., 2018). Interestingly, edaphic specialization and so habitat isolation between E1 and W1 genetic lineages is restricted to the secondary contact zone in southern Belgium. Therefore, pollinator isolation should also be investigated in other secondary contact zones between the two lineages where such a habitat isolation is absent (Martin et al., 2016; Van Rossum et al., 2018), and where other *Hadena* species may feed on *S. nutans* seeds, such as in UK (Young, 1997). In other studies where postzygotically isolated sister taxa occur in sympatry or parapatry, pollinator isolation has been found to be strong (e.g. in *Orchis*, Scopece et al., 2013; and in *Achimenes*, Ramírez-Aguirre et al., 2019) or weak compared to phenological isolation (e.g. in *Gelsemium*, Pascarella, 2007). In contrast, when postzygotic isolation was weak, pollinator isolation between taxa was strong (e.g. in *Mimulus*, Ramsey et al., 2003; in *Costus*, Kay, 2006; and in *Narcissus*, Marques et al., 2007) or moderate (e.g. between *Silene dioica* and *S. latifolia*; Goulson and Jerrim, 1997). The present study provides, to our knowledge, the first example of strongly postzygotically isolated genetic lineages showing an absence of pollinator isolation despite incomplete phenological isolation. However, more studies are needed to understand in which order prezygotic barriers to reproduction tend to arise between plant genetic lineages, and how often they arise due to selection against unfit hybrids (Baack et al., 2015).

Second, there might be facilitation, rather than competition, between the two ecotypes regarding pollination (Ghazoul, 2006; Mitchell et al., 2009; Ha et al., 2021). Indeed, *Hadena albimacula*, the main pollinator of *S. nutans* in southern Belgium, as well as *Coleophora albella*, are rare (De Prins and Steeman, 2021), so that sharing pollinators may represent a mutual plant-pollinator benefit: populations of the two ecotypes together may sustain larger populations of (nursery) pollinators than separately, by offering more nectar resources for adults and more food resources for caterpillars over a longer period (Waser and Real, 1979; Moeller, 2004; Ghazoul, 2005; Tur et al., 2016). The facilitative nature of pollinator sharing would be thus related to the maintenance of a steady pollination service rather than to a greater attractiveness to pollinators (Ghazoul, 2006; Hegland et al., 2009). This better maintenance of pollinator populations over time may be favored given the rarity of *S. nutans* in Belgium and the only partially overlapping flowering periods between ecotypes. The resulting pollination services may be higher and more stable, and overcome the mating costs related to inter-ecotypic pollination. Comparing pollen dispersal patterns for populations of each ecotype in areas where the other ecotype is not present within moth flight distance ability, might contribute to verify whether the occurrence of the two ecotypes of *S. nutans* in parapatry leads to increased pollination services and facilitation.

## Conclusion

The present study on moth pollination is one of the few experiments conducted in natural conditions at landscape scale. Our findings demonstrate the ability of moths, especially of nursery pollinators, to disperse pollen over long distances in natural landscapes, revealing the efficiency of moths –which are declining in Europe (Fox, 2013; MacGregor et al., 2015)– for ensuring gene flow among plant populations. Therefore, our results emphasize the importance of nursery pollinator conservation for population sustainability of the host plant (Aslan et al., 2013). Moreover, there is some evidence of possible seed-predator specificities between the two reproductively isolated genetic lineages of *S. nutans*, and pollinator sharing instead of pollinator isolation when they occur in parapatry, which suggests that conservation of the host plant is also essential for sustaining (rare) pollinator and seed predator communities. This study exemplifies the importance of considering pollination mutualisms in conservation strategies.

## Data availability statement

The datasets presented in this study can be found in online repository at: https://doi.org/10.5281/zenodo.3973097.

## Author contributions

FVR and NN conceived the ideas and designed methodology. CC, FVR and NN carried out field experiments and sampling. CC, with the help of FVR performed dye counting under microscope. CC, with the help of FVR and NN raised the caterpillars. CC viewed the films and analyzed the data. CC led the writing of the manuscript. All authors contributed critically to the article and approved the submitted version.

## Acknowledgments

We thank the Département de la Nature et des Forêts (Service Public de Wallonie), the Cercles des Naturalistes de Belgique and Natagora (Bernard Clesse, Louis-Marie Delescaille, Philippe Gérard, Sébastien Pirotte, Léon Woué) for access to the study sites and authorization to carry out the experiments, Sarah Le Pajolec for fieldwork and germination experiment, Vincent Droissart, Vincent Deblauwe, Tariq Stévart and Marie Savignac for providing us two cameras in 2019 and a manual for building new cameras, Daniel J. Parmentier for camera building and programming and for focus stacking pictures, Stéphane Claerebout (Centre Marie-Victorin) for insect identification, Roxane Beyns for help with GPS data extraction and correction, and geographic distance calculations, Philippe Dubois and Saloua M’Zoudi (Marine Biology Lab, ULB) and Muriel Quinet (ELIA, UCL) for access and help with the fluorescence microscope, Isabelle Mertens and Pierre Talon for help with taking care of the caterpillars, Hélène Martin for providing the distribution map, and Jérémie B. Fant and two reviewers for comments on the manuscript.

## Conflict of interest

The authors declare that the research was conducted in the absence of any commercial or financial relationships that could be construed as a potential conflict of interest.

## Publisher’s note

All claims expressed in this article are solely those of the authors and do not necessarily represent those of their affiliated organizations, or those of the publisher, the editors and the reviewers. Any product that may be evaluated in this article, or claim that may be made by its manufacturer, is not guaranteed or endorsed by the publisher.
