# Supplementary material for: Pollinator sharing between reproductively isolated genetic lineages of Silene nutans
Source: Front Plant Sci. 2022 Oct 21;13:927498. doi: 10.3389/fpls.2022.927498 (PMC9634645; doi:10.3389/fpls.2022.927498)
Supplement: Supplementary file 1 [file DataSheet_1.docx]

**Supplementary material**

**Appendix S1**

To calculate the distances of dye transfers, point coordinates were corrected with RINEX files from ground stations of the WALCORS network (Mariembourg for the Viroin Valley and Werbomont for the Ourthe Valley; Service public de Wallonie, 2020). The resulting horizontal precision was 1.7 ± 1.4 m for the Viroin Valley and 2.0 ± 2.7 m for the Ourthe Valley. For the population Del_Ca, a 50 m tape meter and a laser distance meter (Leica Disto A5) were used to map the individuals due to momentary GPS malfunction. Distances between each recipient individual and each dye source were calculated with the function Add Surface Information, part of the extension 3D Analyst, in ArcGIS Pro v2.4.0 (2019). This function calculates the length of a line in taking topography into account. Topographic information was obtained with the Digital Elevation Model of Wallonia (Service public de Wallonie, 2015).

ArcGIS Pro v.2.4.0. (2019). Esri. [online 22 July 2020] <https://www.esri.com/fr-fr/arcgis/products/arcgis-pro/overview>

Service public de Wallonie. (2015). *Relief de la Wallonie – Modèle Numérique de Terrain (MNT) 2013-2014.* https://geoportail.wallonie.be/catalogue/6029e738-f828-438b-b10a-85e67f77af92.html

Service public de Wallonie. (2020). *Portail Walcors : Réseau permanent de stations GNSS de référence.* <https://gnss.wallonie.be/walcors/stations.html>

**Appendix S2**

To give insight into the nocturnal pollinators of *S. nutans*, observations were made with homemade infrared cameras filming continuously from dusk to dawn (sunset between 21:30 and 22:00, and sunrise between 05:30 and 06:00, UTC+2). Cameras were homemade with a Raspberry Pi connected to a power bank and a lens, inside an airtight plastic box (Figure S2; Appendix S1; Droissart et al., 2021a). Two cameras were used in 2019 (provided by T. Stévart and M. Savignac, ULB) and four new cameras, built according to Droissart et al. (2021b), and slightly modified, in 2020. The modifications allowed for more fine-tuned parametrization of the camera settings, and reduced the risk of overheating (D.J. Parmentier, unpublished data). To detect pollination events, videos were treated with a motion detection software (Janssen, 2018), or entirely watched when there had been too much wind to use the software. In the Ourthe Valley in June 2019, it was too windy for placing a camera in Ham_Si, and Sy_Ca and Coi_Ca were grazed by sheep.

Droissart, V., Azandi, L., Onguene, E. R., Savignac, M., Smith, T. B., & Deblauwe, V. (2021a). PICT: A low-cost, modular, open-source camera trap system to study plant–insect interactions. *Methods in Ecology and Evolution, 12*, 1389–1396. https://doi.org/10.1111/2041-210X.13618

Droissart, V., Azandi, L., Onguene, E. R., Savignac, M., Smith, T. B., & Deblauwe, V. (2021b). How to build and use "PICT"? A users-friendly practical guide (Version 1.0.0). *Methods in Ecology and Evolution*, Zenodo. https://doi.org/10.5281/zenodo.4139839

Janssen, J. P. (2018). *FFmpeg Detect & Copy Motion.* <https://github.com/Jpja/FFmpeg-Detect-Copy-Motion>

**Appendix S3**

Caterpillars and other larvae were reared until reaching adult stage in individual plastic pots to avoid cannibalism (Peschken & Derby, 1990), and fed with capsules of the ecotype on which they were found. Pots were cleaned and a damp paper towel was replaced every day (Figure S3c). For those still at pupal stage at the end of the summer (Figure S3d), pots were placed outside in a garden in Brussels (Belgium), in soil covered with moss until pupae reached adult stage in May-June of the next year and could be identified (Figure S3e).

Peschken, D. P., & Derby, J. L. (1990). Evaluation of *Hadena perplexa* [Lepidoptera: Phalaenidae] as a biocontrol agent of bladder campion *Silene vulgaris* [Caryophyllaceae] in Canada: Rearing and host specificity. *Entomophaga*, *35*, 653–657. https://doi.org/10.1007/BF02375099

**Appendix S4**

To test for differences in dye transfers between populations, a hurdle model was fitted to the sum of dye counts in each region. A hurdle model was chosen due to zero-inflation (excess of zero values in the datasets) and overdispersion in the sum of dye counts (Zuur et al., 2009). The hurdle model (or zero-altered model) is a combination of two distinct models: a binomial Generalized Linear Model (GLM) with logit link that models the probability of dye transfer (presence/absence of dye particles), and a zero-truncated count model (e.g. Poisson or negative binomial) that models the count of dye particles in case of dye presence. This hurdle model thus concurrently analyses two ecological processes: the probability of a dye transfer event (i.e. presence/absence of dye) and the intensity of dye transfer (i.e. dye count) in case of an effective transfer event. A Zero-Altered Negative Binomial (ZANB) model was chosen over a Zero-Altered Poisson (ZAP) model based on a likelihood ratio test (*P* < 0.001) and an AIC comparison (Zuur et al., 2009).

In the Ourthe Valley, a data with an extremely high value was identified as an outlier (Figure S8). As this outlier alone influenced the outcome of the directionality of transfer in the model (significantly higher dye count from Si to Ca populations than the opposite), it was left out from the analysis. To test for a possible effect of using artificial individuals (flowering stalks in pots filled with water) on dye dispersal patterns, an additional analysis was performed by adding the type of recipient plant in Sy_Ca (field plants or flowers in pots) in the hurdle model. The type of recipient plant did not significantly affect the probability of dye transfer (*P* = 0.994 and *P* = 0.078 for intra- and interpopulation transfers, respectively).

To determine whether the higher probability of dye transfers from Ca to Si populations in the Viroin Valley was due to a lack of attractiveness of Del_Ca population, which was small and mainly consisted of gynomonoecious plants, the analysis was performed on the data without the recipient individuals of Del_Ca and the outlier. The results were similar to the analysis including Del_Ca recipient individuals (not shown).

Zuur, A. F., Ieno, E. N., Walker, N. J., Saveliev, A. A., & Smith, G. M. (2009). *Mixed Effects Models and Extensions in Ecology with R*. Springer.

**TABLE S1** Information regarding the eight studied Silene nutans populations from two regions of southern Belgium

| Population | *N* | *n* | S (m²) | Dye source color | Mean distance of recipient individuals to dye source (m) | | | |
| --- | --- | --- | --- | --- | --- | --- | --- | --- |
| Viroin |  |  |  |  | Del_Ca | Rav_Si | Nes_Si | Lom_Ca |
| Del_Ca | 45 | 33 | 6240 | Blue | **53** | 2909 | 3108 | 2673 |
| Rav_Si | 123 | 41 | 7800 | Yellow | 2861 | **91** | 976 | 3416 |
| Nes_Si | 135 | 51 | 15730 | Orange | 3022 | 982 | **98** | 4188 |
| Lom_Ca | 300 | 50 | 4220 | Pink | 2737 | 3392 | 4267 | **42** |
| Ourthe |  |  |  |  | Coi_Ca | Ham_Si | Tom_Si | Sy_Ca |
| Coi_Ca | 120 | 50 | 3602 | Blue | **38** | 2451 | 720 | 2697 |
| Ham_Si | 100 | 33 | 5109 | Yellow | 2409 | **116** | 1724 | 4945 |
| Tom_Si | 70 | 40 | 3125 | Orange | 679 | 1746 | **30** | 3334 |
| Sy_Ca | 41* | 26 | 1122 | Pink | 2717 | 4993 | 3346 | **40** |

*Note:* Mean distances to dye source for intrapopulation transfers are in bold. Columns are source populations, lines are recipient populations. *N* = population size (number of flowering individuals), *n* = number of sampled individuals, S = population area (m²), Ca = calcicolous ecotype, Si = silicicolous ecotype.* In population Sy_Ca, 21 individuals (inflorescences placed in pots with water) were added for the duration of the experiment.

**TABLE S2** Number of flowers per recipient individual and mean number of flowers per inflorescence for each recipient individual (mean value ± SE), for eight calcicolous (Ca) and silicicolous (Si) populations of *Silene nutans* in the Viroin and Ourthe valleys

| Population | Number of flowers per individual | Number of flowers per inflorescence |
| --- | --- | --- |
|  |  |  |
| Viroin |  |  |
| Del_Ca | 48.7 ± 9.2 | 4.7 ± 0.5 |
| Rav_Si | 156.3 ± 20.5 | 8.1 ± 0.4 |
| Nes_Si | 58.8 ± 8.2 | 4.9 ± 0.2 |
| Lom_Ca | 82.8 ± 11.3 | 4.2 ± 0.2 |
| Ourthe |  |  |
| Coi_Ca | 356.7 ± 52.7 | 19.1 ± 1.1 |
| Ham_Si | 409.7 ± 87.7 | 11.4 ± 0.6 |
| Tom_Si | 109.1 ± 15.2 | 7.5 ± 0.3 |
| Sy_Ca* | 32.8 ± 2.7 | 15.1 ± 1.4 |

*Note:* * In population Sy_Ca, most individuals consisted of inflorescences collected in Coi_Ca and placed in pots with water for the duration of the experiment (see Figure S1).

**TABLE S3** Results of cameras filming nocturnal pollinators in eight calcicolous (Ca) and silicicolous (Si) populations of Silene nutans in southern Belgium (mean value ± SE)

| Variable | Population |  | Mann-Whitney *U*-test | |
| --- | --- | --- | --- | --- |
|  | Ca | Si | *U* | *P*-value |
| Number of filmed nights | 6 | 7 | - | - |
| Recording duration (h) | 3.7 ± 0.8 | 5.5 ± 0.8 | 29.5 | 0.252 |
| Number of filmed flowers | 57.7 ± 8.3 | 43.1 ± 8.5 | 12.0 | 0.234 |
| Number of observed moths /h | 0.5 ± 0.2 | 0.9 ± 0.3 | 29.0 | 0.281 |
| Number of visited flowers /h | 0.9 ± 0.6 | 2.0 ± 0.6 | 28.0 | 0.346 |

**TABLE S4** Results of the hurdle model testing for differences between calcicolous (Ca) and silicicolous (Si) populations on the sum of the dye counts per recipient individual for intrapopulation dye transfers (with the number of sampled flowers as an offset, and populations Del_Ca and Coi_Ca as reference populations) for the Viroin and Ourthe valleys. The binomial GLM tested for differences in the probability of dye transfer, while the zero-truncated negative binomial tested for differences in dye counts in case of transfer. Significant Wald tests (Z) are in bold

| Valley | Model part | Regression variables | Coefficient estimate ± SE | *Z* value | *P* value |
| --- | --- | --- | --- | --- | --- |
| Viroin | Binomial GLM | |  |  |  |
|  |  | Total number of flowers | 0.002 ± 0.002 | 1.059 | 0.290 |
|  |  | Distance to dye source | -0.008 ± 0.007 | -1.236 | 0.216 |
|  |  | Rav_Si | 0.995 ± 1.128 | 0.882 | 0.378 |
|  |  | Nes_Si | -0.579 ± 0.775 | -0.747 | 0.455 |
|  |  | Lom_Ca | -0.477 ± 0.896 | -0.532 | 0.595 |
|  |  | Distance to dye source × Rav_Si | -0.002 ± 0.009 | -0.273 | 0.785 |
|  |  | Distance to dye source × Nes_Si | 0.006 ± 0.007 | 0.820 | 0.412 |
|  |  | Distance to dye source × Lom_Ca | -0.025 ± 0.014 | -1.808 | 0.071 |
|  | Zero-truncated negative binomial | |  |  |  |
|  |  | Total number of flowers | 0.001 ± 0.004 | 0.223 | 0.823 |
|  |  | Distance to dye source | -0.033 ± 0.007 | -5.004 | **< 0.001** |
|  |  | Rav_Si | 3.014 ± 1.065 | 2.830 | **0.005** |
|  |  | Nes_Si | -0.070 ± 0.920 | -0.076 | 0.939 |
|  |  | Lom_Ca | -0.180 ± 2.248 | -0.080 | 0.936 |
|  |  | Distance to dye source × Rav_Si | 0.005 ± 0.009 | 0.573 | 0.566 |
|  |  | Distance to dye source × Nes_Si | 0.031 ± 0.008 | 3.827 | **0.001** |
|  |  | Distance to dye source × Lom_Ca | -0.013 ± 0.037 | -0.356 | 0.722 |
| Ourthe | Binomial GLM | |  |  |  |
|  |  | Total number of flowers | 0.001 ± 0.001 | 1.461 | 0.144 |
|  |  | Distance to dye source | -0.031 ± 0.015 | -2.060 | **0.039** |
|  |  | Ham_Si | 1.560 ± 1.486 | 1.049 | 0.294 |
|  |  | Sy_Ca | 16.88 ± 2 338 | 0.007 | 0.994 |
|  |  | Tom_Si | -0.651 ± 1.054 | -0.618 | 0.537 |
|  |  | Distance to dye source × Ham_Si | 0.023 ± 0.016 | 1.446 | 0.148 |
|  |  | Distance to dye source × Sy_Ca | 0.035 ± 28.31 | 0.001 | 0.999 |
|  |  | Distance to dye source × Tom_Si | 0.044 ± 0.028 | 1.599 | 0.120 |
|  | Zero-truncated negative binomial | |  |  |  |
|  |  | Total number of flowers | -0.001 ± 0.001 | -0.665 | 0.506 |
|  |  | Distance to dye source | -0.129 ± 0.025 | -2.169 | **< 0.001** |
|  |  | Ham_Si | -1.130 ± 1.262 | -0.895 | 0.371 |
|  |  | Sy_Ca | -0.373 ± 1.356 | -0.275 | 0.783 |
|  |  | Tom_Si | -2.920 ± 1.696 | -1.722 | 0.085 |
|  |  | Distance to dye source × Ham_Si | 0.118 ± 0.025 | 4.745 | **< 0.001** |
|  |  | Distance to dye source × Sy_Ca | 0.104 ± 0.026 | 4.060 | **< 0.001** |
|  |  | Distance to dye source × Tom_Si | 0.153 ± 0.045 | 3.428 | **0.001** |

**Table S5** Results of the hurdle model testing for differences between intra- and inter-ecotypic dye transfers (type of transfer) on the sum of the dye counts per recipient individual for interpopulation dye transfers (with the number of sampled flowers as an offset) for the Viroin and the Ourthe valleys. The binomial GLM tested for differences in the probability of dye transfer, while the zero-truncated negative binomial tested for differences in dye counts in case of transfer. For the Viroin Valley, an outlier affected the outcome of the model and was thus removed from the analysis. Significant Wald tests (Z) are in bold

| Valley | Model part | Regression variables | Coefficient estimate ± SE | *Z* value | *P* value |
| --- | --- | --- | --- | --- | --- |
| Viroin | Binomial GLM | |  |  |  |
|  |  | Total number of flowers | -0.001 ± 0.001 | -0.873 | 0.383 |
|  |  | Distance to dye source | 0.135 ± 0.182 | 0.743 | 0.458 |
|  |  | Type of transfer | 2.341 ± 0.884 | 2.648 | **0.008** |
|  |  | Distance to dye source × type of transfer | -0.870 ± 0.296 | -2.938 | **0.003** |
|  | Zero-truncated negative binomial | |  |  |  |
|  |  | Total number of flowers | 0.001 ± 0.002 | 0.087 | 0.931 |
|  |  | Distance to dye source | -0.474 ± 0.293 | -1.619 | 0.105 |
|  |  | Type of transfer | 2.247 ± 1.425 | 1.577 | 0.115 |
|  |  | Distance to dye source × type of transfer | -0.576 ± 0.479 | -1.204 | 0.229 |
| Ourthe | Binomial GLM | |  |  |  |
|  |  | Total number of flowers | 0.001 ± 0.001 | 0.537 | 0.592 |
|  |  | Distance to dye source | 1.042 ± 0.361 | 2.885 | **0.004** |
|  |  | Type of transfer | 1.348 ± 0.849 | 1.587 | 0.112 |
|  |  | Distance to dye source × type of transfer | -0.907 ± 0.370 | -2.453 | **0.014** |
|  | Zero-truncated negative binomial | |  |  |  |
|  |  | Total number of flowers | 0.001 ± 0.001 | 2.154 | **0.031** |
|  |  | Distance to dye source | 1.547 ± 0.636 | 2.432 | **0.015** |
|  |  | Type of transfer | 4.112 ± 1.416 | 2.903 | **0.004** |
|  |  | Distance to dye source × type of transfer | -1.657 ± 0.633 | -2.620 | **0.009** |

**Table S6** Results of the hurdle model testing for differences between transfers from silicicolous to calcicolous populations and the opposite (type of transfer) on the sum of the dye counts per recipient individual for inter-ecotypic dye transfers (with the number of sampled flowers as an offset) for the Viroin and the Ourthe valleys. The binomial GLM tested for differences in the probability of dye transfer, while the zero-truncated negative binomial tested for differences in dye counts in case of transfer. For the Ourthe Valley, an outlier affected the outcome of the model and was thus removed from the analysis. Significant Wald tests (Z) are in bold

| Valley | Model part | Regression variables | Coefficient estimate ± SE | *Z* value | *P* value |
| --- | --- | --- | --- | --- | --- |
| Viroin | Binomial GLM | |  |  |  |
|  |  | Total number of flowers | -0.001 ± 0.001 | -0.050 | 0.960 |
|  |  | Distance to dye source | -0.105 ± 0.350 | -0.300 | 0.764 |
|  |  | Type of transfer | 4.478 ± 1.678 | 2.669 | **0.008** |
|  |  | Distance to dye source × type of transfer | -1.153 ± 0.492 | -2.345 | **0.019** |
|  | Zero-truncated negative binomial | |  |  |  |
|  |  | Total number of flowers | -0.002 ± 0.003 | -0.772 | 0.440 |
|  |  | Distance to dye source | -0.762 ± 0.604 | -1.261 | 0.207 |
|  |  | Type of transfer | 3.454 ± 2.768 | 1.248 | 0.212 |
|  |  | Distance to dye source × type of transfer | -0.862 ± 0.828 | -1.041 | 0.298 |
| Ourthe | Binomial GLM | |  |  |  |
|  |  | Total number of flowers | -0.001 ± 0.001 | -1.305 | 0.192 |
|  |  | Distance to dye source | -0.036 ± 0.125 | -0.291 | 0.771 |
|  |  | Type of transfer | 0.418 ± 0.514 | 0.814 | 0.415 |
|  |  | Distance to dye source × type of transfer | 0.256 ± 0.174 | 1.468 | 0.142 |
|  | Zero-truncated negative binomial | |  |  |  |
|  |  | Total number of flowers | 0.001 ± 0.001 | 1.322 | 0.186 |
|  |  | Distance to dye source | -0.248 ± 0.366 | -0.677 | 0.499 |
|  |  | Type of transfer | -1.464 ± 1.143 | -1.280 | 0.200 |
|  |  | Distance to dye source × type of transfer | 0.561 ± 0.399 | 1.406 | 0.160 |

**TABLE S7** Seed germination rate for eight calcicolous (Ca) and silicicolous (Si) populations of *Silene nutans* from southern Belgium. Chlorotic (yellow) or partially chlorotic (light green) individuals likely corresponded to inter-ecotypic hybrids

| Population | Seed number | Germination (%) | Hybrids (%) |
| --- | --- | --- | --- |
| Viroin |  |  |  |
| Del_Ca | 100 | 63 | 3.2 |
| Lom_Ca | 200 | 84 | 2.4 |
| Rav_Si | 100 | 94 | 1.1 |
| Nes_Si | 100 | 95 | 1.1 |
| Ourthe |  |  |  |
| Coi_Ca | 100 | 98 | 4.1 |
| Sy_Ca | 100 | 82 | 0.0 |
| Ham_Si | 100 | 95 | 0.0 |
| Tom_Si | 200 | 88 | 1.7 |

**(a) Viroin Valley (b) Ourthe Valley**

**
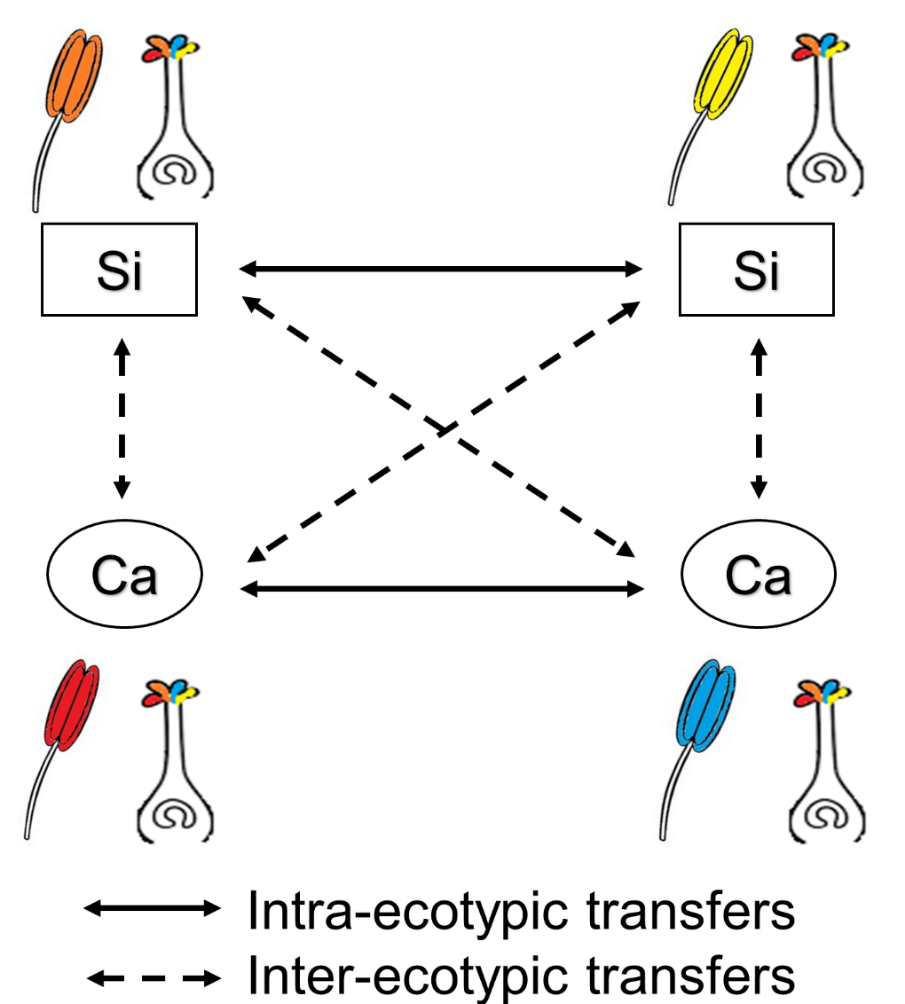

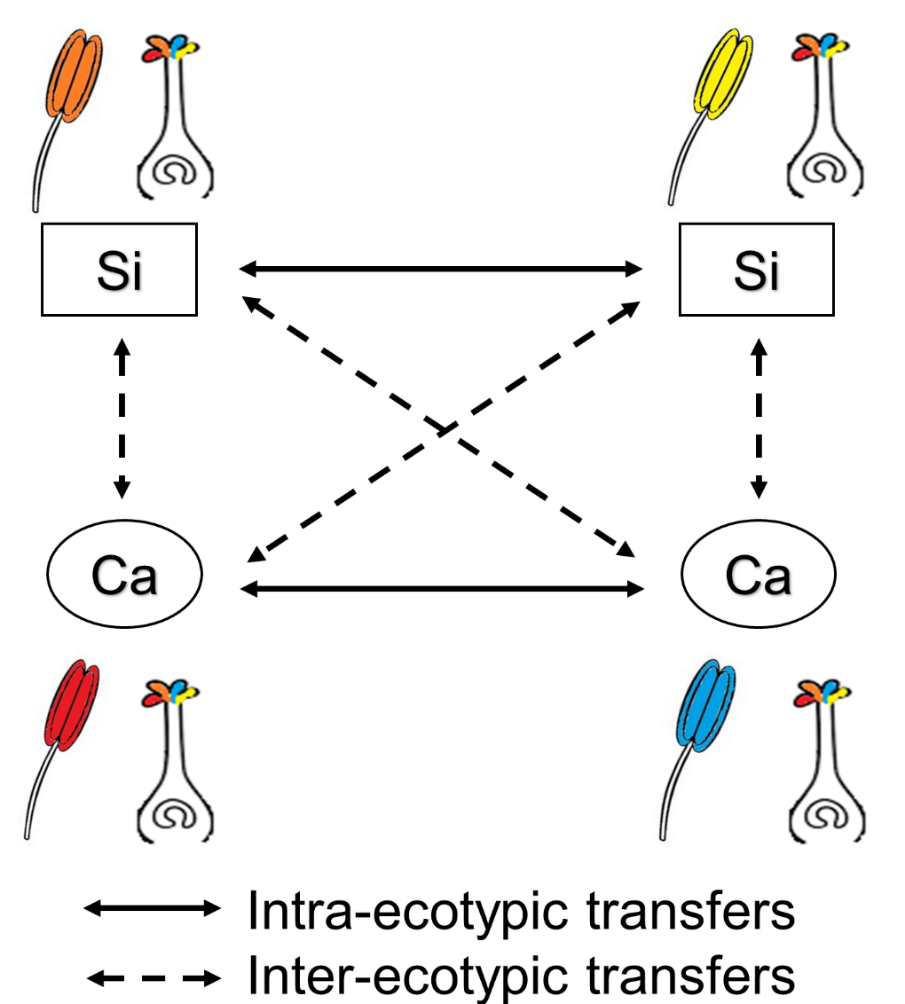
**

53.8-78.0

27.5-60.6

24.0-84.8

39.4-48.0

22.0-49.0

13.7-54.9

**FIGURE S1** Experimental design for testing for pollinator isolation or sharing between populations of the calcicolous (Ca) and silicicolous (Si) ecotypes of *S. nutans*, by comparing intra- and inter-ecotypic dye transfers, for (a) the Viroin and (b) Ourthe valleys, with the percentage of recipient individuals showing dye deposition (range of mean values per population; for more details, see Table 1).


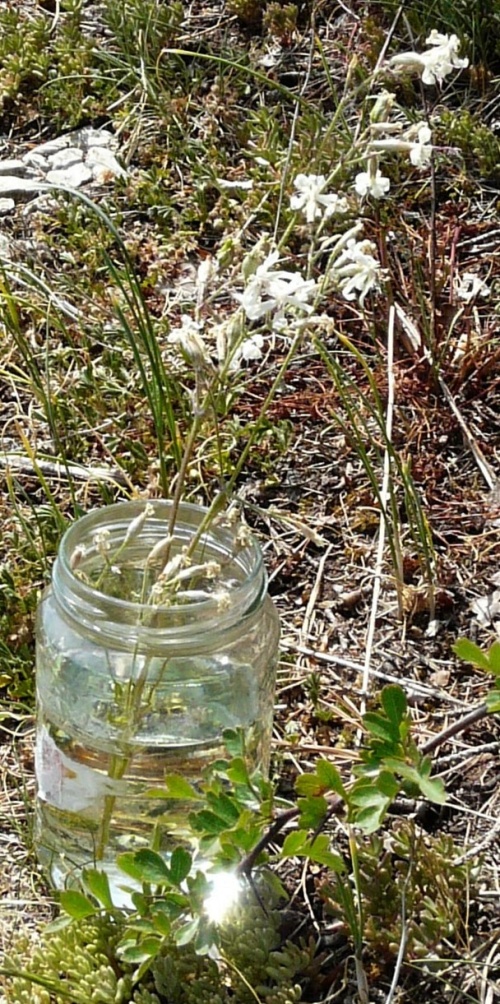


**FIGURE S2** One of the 21 artificial individuals of S. nutans added to population Sy_Ca (Picture: F. Van Rossum).


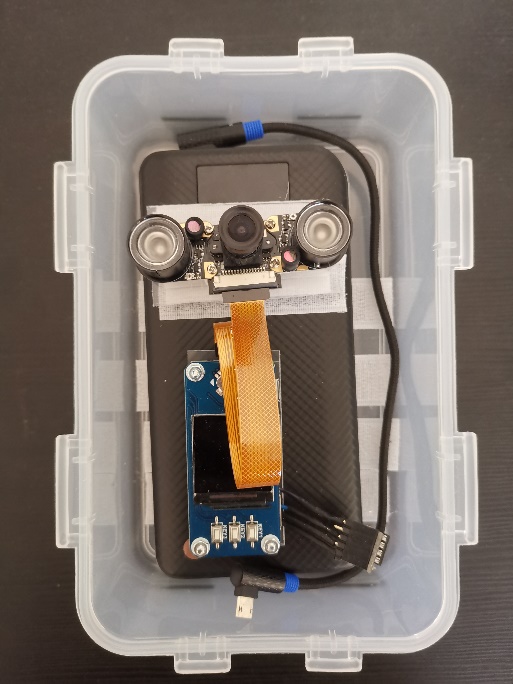

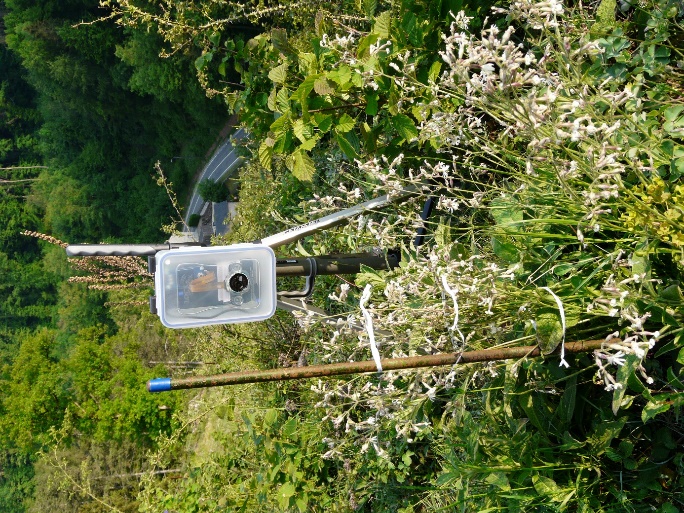


**FIGURE S3** Homemade camera with a Raspberry Pi and camera fixed on a tripod in the field in front of the dye source individuals (Pictures: C. Cornet, F. Van Rossum).


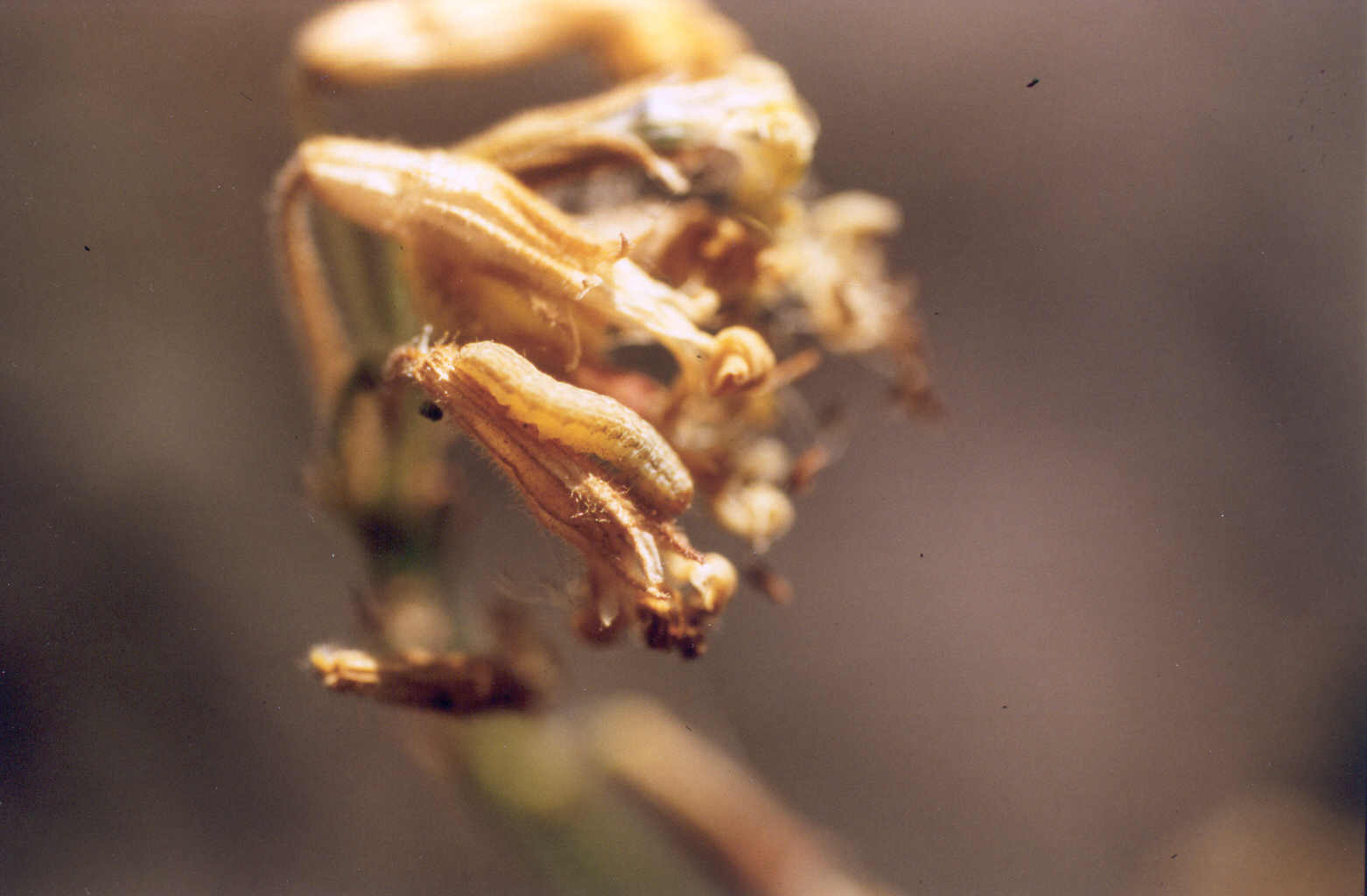

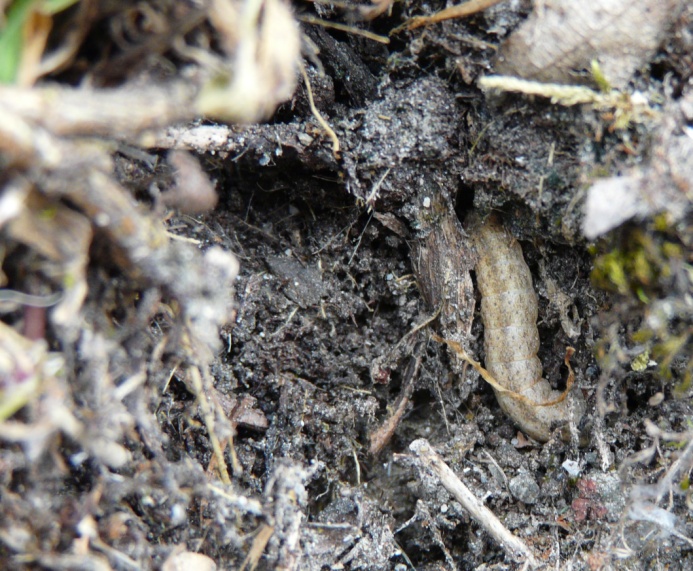


(b)

(a)


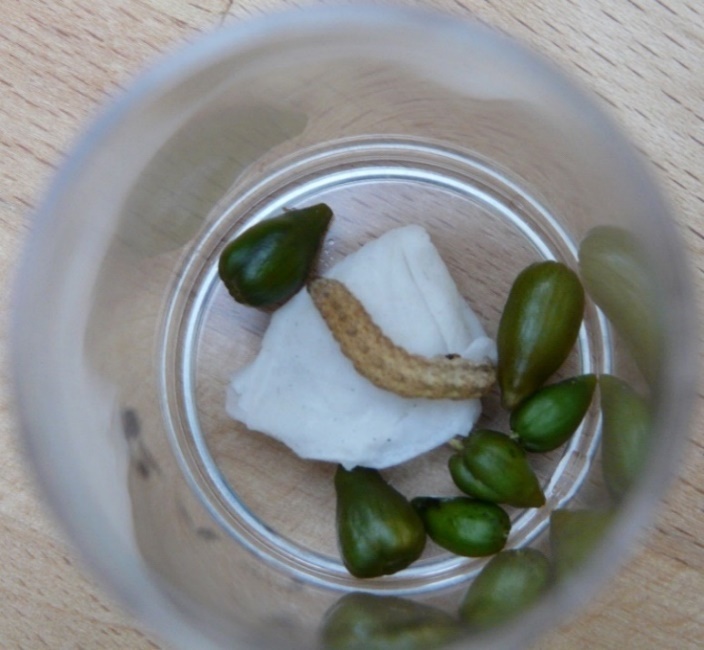

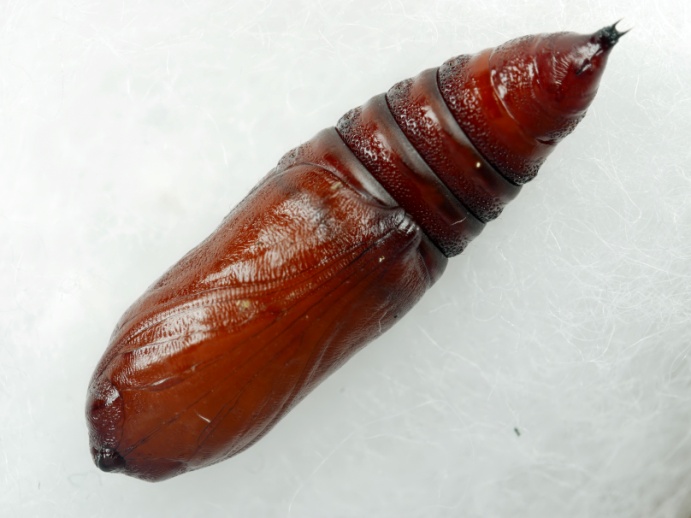


(d)

(c)


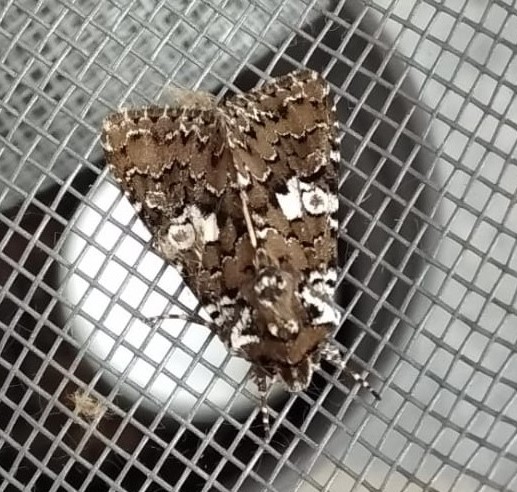


(e)

**FIGURE S4** Hadena albimacula (nursery pollinator of Silene nutans): caterpillar (a) on S. nutans flowers and (b) hidden under a plant in the field; and (c) during rearing, with green capsules of S. nutans as food; (d) pupa and (e) emerging adult (Pictures: (a-c) F. Van Rossum; (d) D.J. Parmentier; (e) N. Noret).


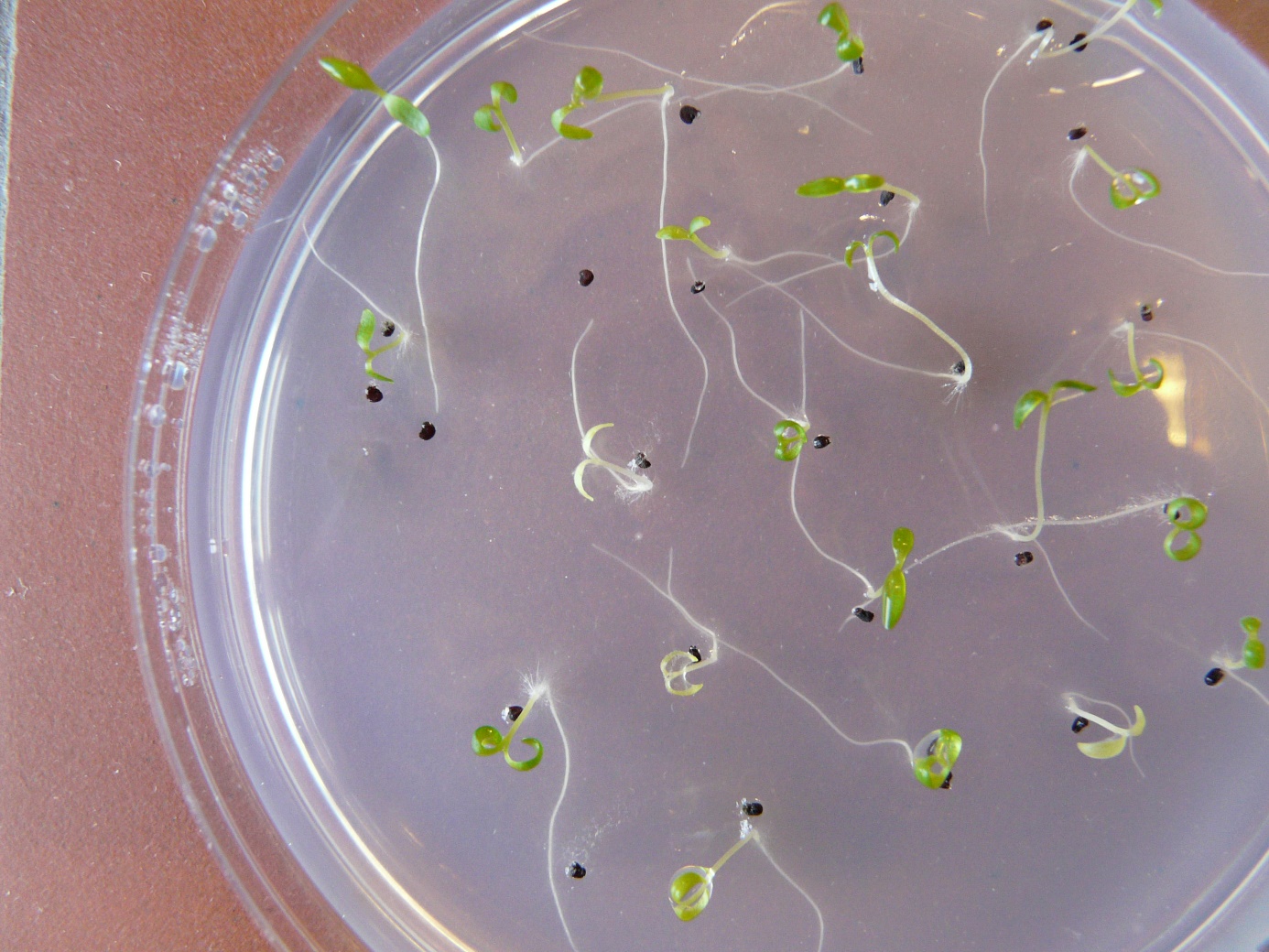


a

b

**FIGURE S5** Germinated seedlings of Silene nutans (from Coi_Ca population): (a) chlorotic hybrid, (b) healthy seedling (Picture: F. Van Rossum).

**
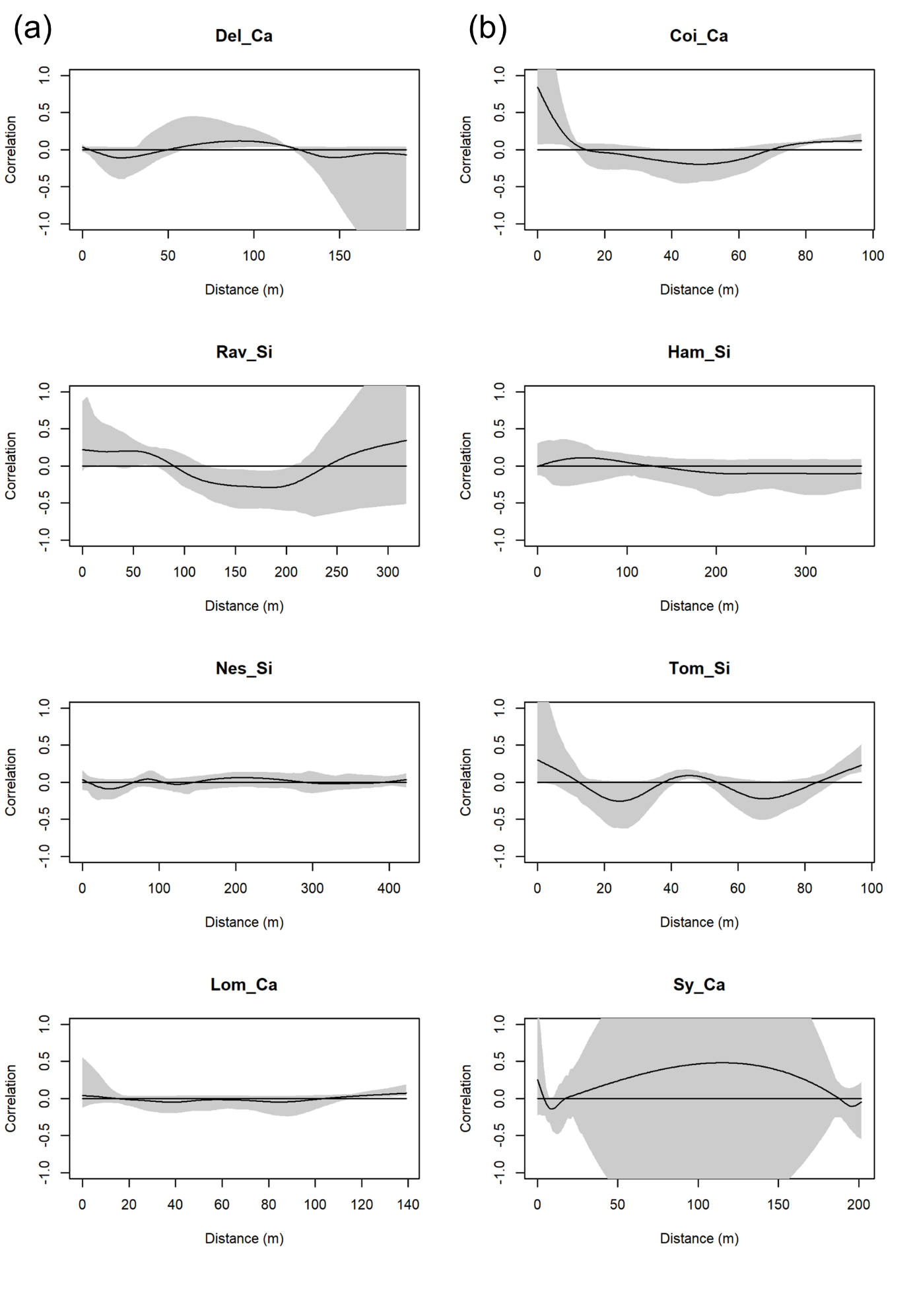
**

**FIGURE S6** Spatial spline autocorrelograms of intrapopulation dye transfers in (a) the Viroin Valley and (b) the Ourthe Valley.

**
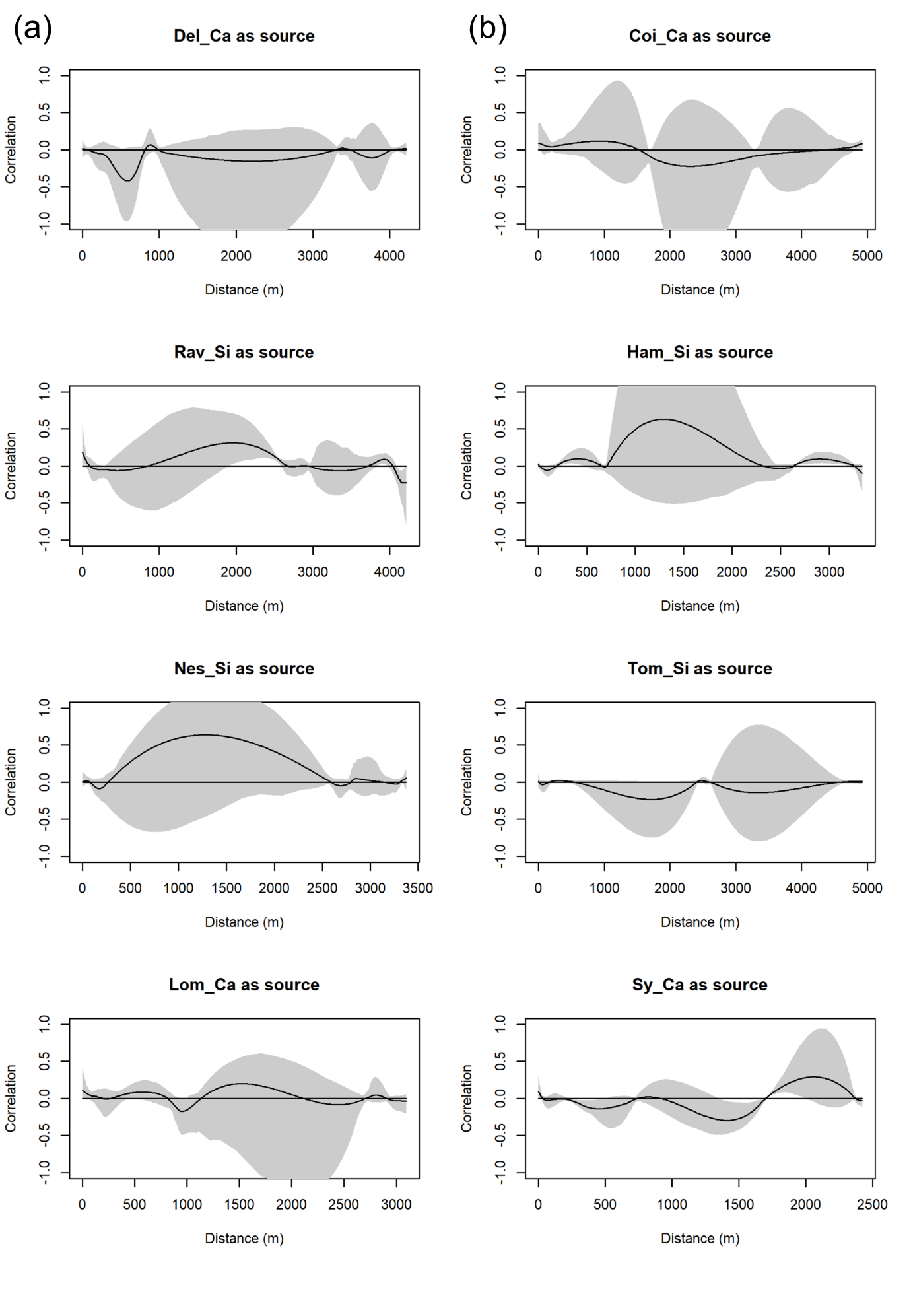
**

**FIGURE S7** Spatial spline autocorrelograms of interpopulation dye transfers in (a) the Viroin Valley and (b) the Ourthe Valley, separately for each dye source population.


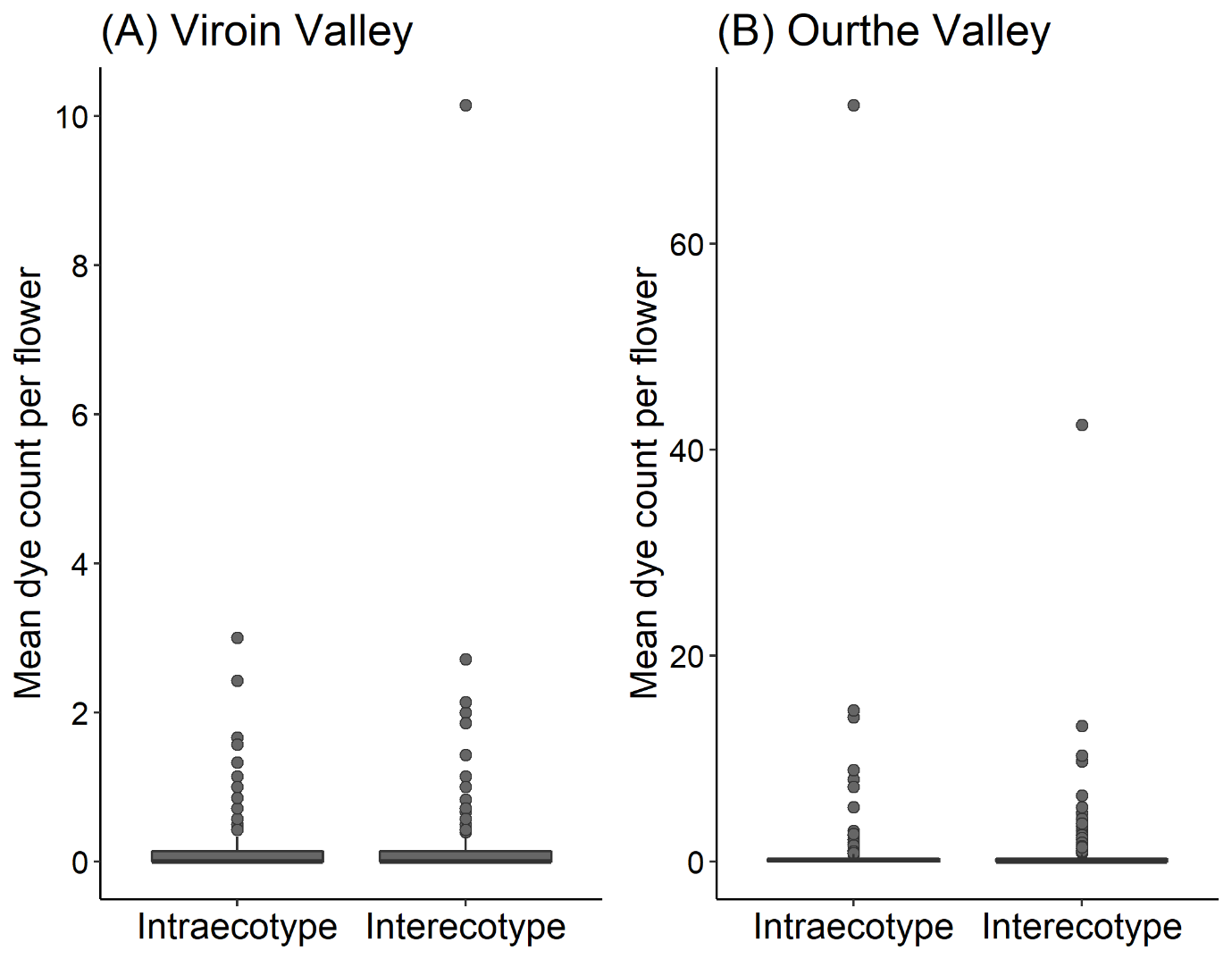


**FIGURE S8** Boxplots of the mean dye count per flower per recipient individual and per dye source in function of the type of transfer (inter-ecotypic or intra-ecotypic) in (a) Viroin and (b) Ourthe Valley.


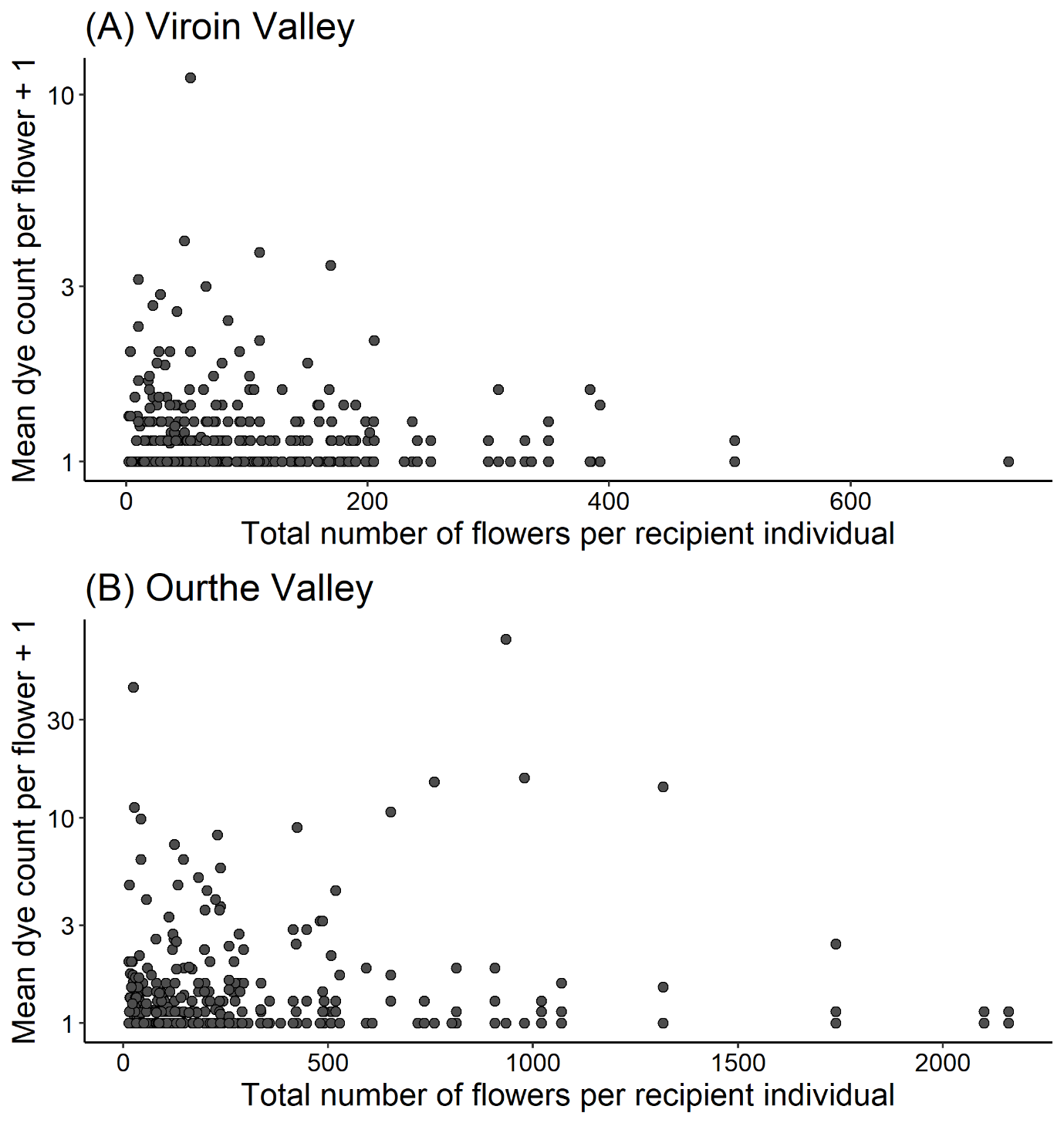


**FIGURE S9** For interpopulation dye transfers, mean dye count per flower in function of total number of flowers per recipient individual, in (a) Viroin and (b) Ourthe Valley.
